# A Dispensable Chromosome Is Required for Virulence in the Hemibiotrophic Plant Pathogen *Colletotrichum higginsianum*

**DOI:** 10.3389/fmicb.2018.01005

**Published:** 2018-05-18

**Authors:** Peter-Louis Plaumann, Johannes Schmidpeter, Marlis Dahl, Leila Taher, Christian Koch

**Affiliations:** ^1^Division of Biochemistry, Department of Biology, Friedrich-Alexander-Universität Erlangen-Nürnberg, Erlangen, Germany; ^2^Division of Bioinformatics, Department of Biology, Friedrich-Alexander-Universität Erlangen-Nürnberg, Erlangen, Germany

**Keywords:** Arabidopsis, plant defense, protein effectors, chromosome loss, B-chromosomes, virulence

## Abstract

The hemibiotrophic plant pathogen *Colletotrichum higginsianum* infects *Brassicaceae* and in combination with *Arabidopsis thaliana*, represents an important model system to investigate various ecologically important fungal pathogens and their infection strategies. After penetration of plant cells by appressoria, *C. higginsianum* establishes large biotrophic primary hyphae in the first infected cell. Shortly thereafter, a switch to necrotrophic growth occurs leading to the invasion of neighboring cells by secondary hyphae. In a forward genetic screen for virulence mutants by insertional mutagenesis, we identified mutants that penetrate the plant but show a defect in the passage from biotrophy to necrotrophy. Genome sequencing and pulsed-field gel electrophoresis revealed that two mutants were lacking chromosome 11, encoding potential pathogenicity genes. We established a chromosome loss assay to verify that strains lacking this small chromosome abort infection during biotrophy, while their ability to grow on artificial media was not affected. *C. higginsianum* harbors a second small chromosome, which can be lost without effects on virulence or growth on agar plates. Furthermore, we found that chromosome 11 is required to suppress *Arabidopsis thaliana* plant defense mechanisms dependent on tryptophan derived secondary metabolites.

## Introduction

Pathogenic fungi employ different strategies for causing disease. Biotrophic fungi like the rusts or powdery mildew fungi show a narrow host range and highly adapted genomes showing gene expansions of pathogenicity related genes while having a reduced repertoire of genes involved in primary and secondary metabolism. This specializes them to keep the infected host cells alive and suppress plant defense (Spanu et al., 2010; Duplessis et al., 2011; Raffaele and Kamoun, 2012). Extra- and intracellular effector proteins play a central role in regulating these interactions (Stergiopoulos and de Wit, 2009). Both effector-triggered susceptibility and effector-triggered immunity leave their marks in the genomes of pathogenic fungi which often show species-specific gene duplications and genome expansions involving effector functions required for virulence (Raffaele and Kamoun, 2012; Jones et al., 2016). Some effectors have been genetically identified and the recent genome analysis of many filamentous pathogens has potentially provided the full repertoire of virulence genes and effectors proteins (Raffaele and Kamoun, 2012; Moller and Stukenbrock, 2017). However, genetic insights into the function of individual pathogenicity determinants are frequently lacking because loss of function mutants often show no pathogenicity phenotype. This is usually attributed to functional redundancy of effector gene functions (Koeck et al., 2011).

The genomic analysis of fungal and oomycete pathogens has also led to the identification of genomic regions encoding high numbers of potential effector genes (Dong et al., 2015). Such regions, like the pathogenicity related gene clusters in *Ustilago maydis* (Kamper et al., 2006), are often sparsely covered with housekeeping genes (Raffaele and Kamoun, 2012). *Magnaporthe* strains, for example, may differ in the presence or absence of regions as large as 1.7 mb (Yoshida et al., 2009), while *Verticillium dahliae* contains a 4 mb lineage specific region (de Jonge et al., 2013). In the case of cereal rust fungi it was shown that a 2.5 mb large genomic region harboring Avr genes can be altered by unequal recombination (Chen et al., 2017), not only leading to genetic variations but also as a mechanism for breaking resistance. Chromosomal variations have been particularly well documented by comparative genomics (Dong et al., 2015) and are reflecting the adaptation to environmental cues and host defense mechanisms (Seidl and Thomma, 2014; Jones et al., 2016). Such regions of chromosomal polymorphisms can be very large and may even encompass whole chromosomes as in the case of *Fusarium* pathogens that harbor host range specific chromosomes (Ma et al., 2010). Such chromosomes may even become dispensable for growth under saprophytic conditions (Beadle et al., 2003) as is seen in *Alternaria* (Tsuge et al., 2013), *Nectria haematococca* (VanEtten et al., 1998) or in *Leptosphaeria maculans* (Balesdent et al., 2013). Dispensable chromosomes or supernumerary chromosomes, often also referred to as B-chromosomes (Jones, 1991) and originally known from the analysis of the rye plant genome, may be a hallmark of quickly evolving genomes (Camacho et al., 2000). 14 species of fungi have been reported to contain dispensable chromosomes (D’Ambrosio et al., 2017) including *Colletotrichum gloeosporioides* (Masel et al., 1996). A role for these in pathogenicity has not been identified in *Colletotrichum* species so far. The recent analysis of the *C. higginsianum* genome, in particular the assembly of complete chromosomes (O’Connell et al., 2012; Dallery et al., 2017), now allows to map virulence functions to individual genomic regions. *C. higginsianum* harbors 12 chromosomes, two much smaller than the others with less than 1000 kb DNA in size.

*Colletotrichum higginsianum* pursues a hemibiotrophic lifestyle (O’Connell et al., 2004). After penetration of the host plant with the help of appressoria, it forms primary biotrophic hyphae which have a characteristic large and bulbous appearance. The biotrophic stage during the infection in which the host cell remains alive, is confined to the first infected cell. After 2–3 days the pathogen forms filamentous secondary hyphae and switches its lifestyle and nutritional strategy. Then, necrotrophic hyphae colonize and kill the neighboring tissue (O’Connell et al., 2012). The switch to necrotrophic growth is accompanied with characteristic changes in gene expression and with the induction of genes encoding specific effector proteins (Kleemann et al., 2012). The signals orchestrating this developmental switch are largely unknown.

Together with *Arabidopsis thaliana*, *C. higginsianum* is a prominent model system for the analysis of hemibiotrophic plant pathogen interactions (O’Connell and Panstruga, 2006). Forward genetic screens, expression analysis and functional characterization of candidate genes have identified an array of pathogenicity determinants and effectors in *C. higginsianum* (Kleemann et al., 2008, 2012; Huser et al., 2009; O’Connell et al., 2012; Liu et al., 2013; Korn et al., 2015; Schmidpeter et al., 2017). We have previously isolated a series of *Colletotrichum higginsianum* mutants with different characteristic virulence defects on the host plant *Arabidopsis thaliana* (Korn et al., 2015; Schmidpeter et al., 2017). Some mutants penetrated the host successfully but were arrested during the biotrophic phase of infection. For two of these virulence mutants, we here report that the defect is due to the loss of the small chromosome 11. *Colletotrichum higginsianum* harbors two small chromosomes. Whereas chromosome 11 is critically important for virulence, we find that chromosome 12 can be lost without affecting pathogenicity.

## Materials and Methods

### Fungal and Bacterial Strains

*Agrobacterium tumefaciens* strain AGL1 (AGLO recA::bla pTiBo542ΔT Mop+ CbR) was used for fungal transformation as described (Korn et al., 2015). *Colletotrichum higginsianum* WT strain MAFF 305635 (O’Connell et al., 2004) (CY7444) was from the Ministry of Agriculture, Forest and Fisheries collection, Japan. All other *C. higginsianum* (*Ch*) strains used in this study are listed in the Supplementary Data S1.

### Arabidopsis Lines

*Arabidopsis thaliana* Col-0 was obtained from the local seed collection. Mutant *A. thaliana* lines were congenic to Col-0 unless specifically noted. *pen2-2* (Bednarek et al., 2009) was obtained from P. Schulze-Lefert (Cologne). All other mutant lines were obtained from L. Voll (Marburg). The *cyp79b2/cyp79b3* double mutant (Zhao et al., 2002) was originally obtained from Tamara Gigolashvili. *pad3-1* was originally obtained from Jane Glazebrook (Glazebrook et al., 1997). *NahG* (Lawton et al., 1995) was originally obtained from Yves Marco. *sid2* was obtained from the NASC seed collection (N542603, SALK_042603) and screened for homozygosity. *cerk1-2* (Miya et al., 2007) was obtained from the GABI-KAT seed collection (GK-096F09) and screened for homozygosity. The starch free *p-pgm* mutant line was originally obtained from Caroline Dean and was in the *Ler-0* background.

### Media and Cultivation Methods

*Escherichia coli*, *A. tumefaciens*, and *C. higginsianum* strains were cultured and transformed as described (Korn et al., 2015). For the selection of fungal transformants nourseothricin was used at 100 μg/ml. Hygromycin was used at 72 U/ml. Bialaphos-resistant transformants were selected on Czapek Dox plates supplemented with 2.64 g/l ammonium sulfate and were containing 20 μg/ml bialaphos. 100 μg/ml cefotaxime was added to transformation plates for selecting against *A. tumefaciens. A. thaliana* plants were grown for 2 weeks under short-day conditions (8 h of light, 22°C/16 h of dark, 20°C) and then transferred to growth chambers with 12 h light (22°C)/12 h dark (19°C) cycles (120 μE/m^2^s; 70% humidity). After 3 weeks (5 weeks in total), the plants were infected with *C. higginsianum*. For quantification of fungal biomass in *Arabidopsis* mutant lines, plants were grown for 1 additional week at 12 h light/12 h dark (6 weeks in total). Infections with *C. higginsianum* and induction of appressoria were performed as described (Schmidpeter et al., 2017). For all transformed *C. higginsianum* strains 2–4 independent original transformants were analyzed.

### Standard Techniques and DNAs

DNA manipulations, PCR reactions and plasmid DNA isolations followed standard protocols as described (Korn et al., 2015). Plasmid constructions and oligonucleotide sequences are listed in the Supplementary Data S1.

### Microscopy and Histochemical Staining

Histochemical samples or samples containing fluorescent reporter proteins were stained and analyzed by microscopy as described (Korn et al., 2015) with the following modifications. Quantification of trypan blue stained fungal infection structures was performed with 2 infected plants per strain at 3 dpi. Three leaves were analyzed per plant. At least 600 appressoria were counted per leaf. For the analysis of *in planta* appressoria formation, calcofluor staining was performed 18 h post infection. Leaf sections were cut out without the midrib and placed on the abaxial surface on ethanol soaked whatman paper (3 MM) to remove chlorophyll. After 60 min, the sections were placed on water soaked whatman paper and incubated for additional 20 min. Subsequently, sections were placed on glass slides and 100 μl calcofluor-white solution was added (0.1 mg/ml). Epifluorescence was detected with an excitation band pass filter (340–380 nm) and observed with a long pass filter (LP425 nm). Quantification was performed with 2 sections of 2 infected plants per strain. Detection of reactive oxygen species was performed as described (Korn et al., 2015) with the following modifications. Two leaves each of two infected *A. thaliana* plants per fungal strain were harvested after 4 days. The staining solution was supplemented with 0.05% (v/v) Tween 20. After 18 h incubation in the dark, the staining solution was replaced by destaining solution (ethanol:acetic acid:glycerol = 3:1:1) and boiled for 15 min at 95°C. After mounting with water, the leaves were viewed with bright-field microscopy.

### Pulsed-Field Gel Electrophoresis (PFGE)

Pulsed-field gel electrophoresis was performed on a Hoefer HG 1000 HULA unit using 1% agarose (Agarose Pulsed-Field, Roth) and 0.5x TBE buffer. Gels were pre-run at 10°C for 2 h at 140 V without alternating the electric field, then for 36 h with alternating angles of 120°. The switch time for alternating the electric field was increased from 60 to 120 s over the course of the run. Yeast CHEF DNA size markers (BioRad) were used as standards. For documentation, gels were stained with ethidium bromide (0.5 mg/l) for 30 min. Plugs containing fungal conidia were prepared following published protocols (Maringele and Lydall, 2006). Freshly grown conidia were suspended in water containing 0.005% NaN_3_ and washed twice with H_2_O afterward. 1 × 10^7^ conidia were collected by centrifugation, resuspended in 300 μl SCE buffer (1 M sorbitol, 100 mM sodium-citrate, 60 mM EDTA, pH 7, 60 mM 2-mercaptoethanol) and incubated at 37°C. After adding 50 μl enzyme solution [10 mg/ml Zymolyase T20 (Seikagaku) in SCE buffer], cells were gently mixed with 350 μl low melting point agarose (Bio&Sell) preheated to 50°C. After pipetting up and down twice, the mixture was spread on a casting form, generating 1.5 mm thick plugs. Plugs cut to the appropriate size were incubate in 400 μl SCE with 10 mg/ml Zymolyase T20 for 2 h at 37°C. After washing twice in PFGE buffer (10 mM pH 7.6, 50 mM EDTA) and once in PK buffer (100 mM EDTA, 0.2% sodium desoxycholate, 1% sodium *N*-laurylsarcosine, pH 8), plugs were incubated in 400 μl PK buffer containing 1 mg/ml Proteinase K at 50°C overnight (18 h). The following day, plugs were washed twice in PFGE buffer and stored in the buffer at 4°C until use.

### DNA Sequencing

Genomic DNA sequencing was performed by a commercial provider using chromosomal DNA from *Colletotrichum higginsianum* strain MAFF 305635 and the mutant derivatives *vir-49* and *vir-51*. High molecular weight DNA was prepared as described (Korn et al., 2015). Genomic sequencing was performed on an Illumina MiSeq platform using DNA fragmented to a size between 600 and 900 bp. The raw read files (.fastq accessions: SAMN08226879, SAMN08226880, SAMN08226881) underwent quality control analysis using FastQC.^1^ The quality-checked forward and reverse reads were trimmed separately with cutadapt (v1.14)^2^ with following parameters: -u 5 -u -5 -q 25 –trim-n –max-n 5 –minimum-length = 80 (forward reads) and -u 5 -u -25 -q 25 –trim-n –max-n 5 –minimum-length = 80 (reverse reads). Only read pairs where both the forward and reversed read fulfilled the criteria were kept for further analysis. Reads were aligned as single reads using Bowtie2 (v2.2.9) (Langmead and Salzberg, 2012) with default parameters to the reference assembly of strain IMI 349063 (accession: GCA_001672515.1) and deposited into the sequence read archive (SRA) at NCBI (accession number of bioproject: PRJNA427316). Raw library sizes were 13,696,134 for WT, 7,246,183 for *vir-49* and 6,367,668 for *vir-51*. Read lengths were 266 bp for WT, 250 bp for *vir-49*, and 259 bp for *vir-51*. This corresponds to a coverage of approximately 72x for WT, 36x for *vir-49* and 33x for *vir-51* (Supplementary Tables S1, S2). For further analysis see the Supplementary Data S2.

### Transformation Protocol for the Selection of Homologous Recombinants

*Colletotrichum higginsianum* strain MAFF 305635 was transformed as described (Korn et al., 2015) with the following modifications to identify homologous recombinants. Transformation plates were incubated for 16 days to induce sufficient conidiation on the primary transformation plates. Conidia were rinsed off the transformation plates with 3 ml H_2_O and further propagated on oatmeal-agar (OMA) plates for 1 week to generate a population of transformed conidia. The conidia were again rinsed off with water and diluted to 3 × 10^2^ spores/ml. Hundred microliter of this dilution (*n* = 30 cells) was plated on minimal medium plates (20 μg/ml bialaphos and 100 μg/ml cefotaxime). Two days later, single colonies were picked and further propagated on 12-well OMA plates for 6 days. Subsequently, individual clones were analyzed on antibiotic containing media to identify transformants having undergone homologous recombination which should be resistant to bialaphos but sensitive to nourseothricin (Supplementary Figure S1). To verify homologous recombination, DNAs from such transformants were tested by PCR using primers diagnostic for integration in chromosome 11 (3′ homology region: CK3831/CK5328; 5′ homology region: CK4042/CK5327) or for chromosome 12 (3′ homology region: CK3831/CK5330; 5′ homology region: CK4042/CK5329).

### Sorting of mCherry Negative Cells

Strains expressing cytosolic mCherry from a reporter gene inserted on either chromosome 11 (*chr11::mCherry)* or chromosome 12 (*chr12::mCherry*) were grown for 10 days on OMA plates under standard conditions and then kept at 8°C for 1 day. Prior to FACS analysis, cells were stained with the vital dye CellTrace CFSE (Thermo Fisher, C34570). 5 × 10^7^ conidia were dissolved in 1 ml staining solution (1:1000 dilution in 1xPBS). After 20 min in the dark, 5 ml 1xPBS 1% BSA were added. After 5 additional minutes, conidia were spun down at 2000 rpm for 2 min and resuspended in 1 ml PBS. Prior to flow cytometry, the cell suspension was diluted 10-fold. Cell sorting was performed using a Beckman & Coulter Astrios platform. Sorted cells (mCherry negative conida) were plated out on PDA plates without antibiotics. After 2 days, single colonies were isolated and propagated on 12-well OMA plates for 7 days. To test for the presence of the particular chromosome, we isolated genomic DNA as described (Korn et al., 2015) and conducted PCR for genes located on chromosome 11 (CK5230/CK5231 and CK5192/CK5193) or chromosome 12 (CK5196/CK5197 and CK5304/CK5305), respectively. To validate the result, we performed pulsed-field gel electrophoresis as described above.

### Assessment of Fungal Proliferation in Plant Tissue via Quantitative PCR

To assess fungal proliferation in *Arabidopsis* leaves, we performed quantitative PCR on fungal genomic DNA. For each fungal strain, we infected 5 plants. After 90–96 h, 5 samples per fungal strain were taken by pooling leaf punches from different plants, harvesting a total of 1.5 cm^2^ of infected leaf area per sample in liquid nitrogen. To extract total genomic DNA, we used the Qiagen DNeasy Kit. Quantification of fungal DNA was subsequently analyzed with an Agilent AriaMX Real-Time PCR System using Promega’s GoTaq^®^ qPCR Master Mix and primers for the *C. higginsianum* TrpC gene (Ch TrpC fw and ChTrpC rev). All samples were measured as 3 technical replicates. The relative quantity of fungal DNA was calculated as described (Rieu and Powers, 2009) and normalized to the leaf sample area.

### Extraction of Total RNA From Infected Plant Tissue

For the extraction of total RNA from infected plant tissue, three samples were harvested for each fungal strain and frozen in liquid nitrogen. For each sample three fully expanded leaves from independent plants were pooled and ground up in liquid nitrogen. For the isolation of total RNA, leaf tissue was frozen and ground in liquid nitrogen. The resulting powder was suspended in 3 ml of RNA isolation working solution (4 M guanidinium thiocyanate, 25 mM sodium acetate, 0.5% *N*-lauroylsarcosine, 0.7% mercaptoethanol, pH 7, freshly mixed with equal amounts of water saturated phenol). After thawing, the homogenate was transferred to two reaction tubes (2 ml) each containing 0.3 ml of chloroform:isoamylalcohol (24:1) and vortexed. After incubation for 30 min on ice, tubes were centrifuged for 15 min at 11.000 rpm and 4°C. The aqueous phase was extracted once more with phenol (0.7 ml phenol + 0.3 ml chloroform:isoamylalcohol) and once with chloroform:isoamylalcohol. Subsequently the volume of the upper aqueous phase was determined, 1/20 volume 1 M acetic acid and 1 volume of ethanol was added. After mixing, the pecipitated RNA was collected by centrifugation for 8 min at 13.000 rpm and 4°C. The supernatant was removed and the resulting pellet was washed with 1 ml 3 M sodium acetate pH 6. The two pellets of each sample were combined into one reaction tube by pipetting and pelleted for 10 min at 13.000 rpm and 4°C. The resulting pellet was washed twice with 80% ethanol. The RNA pellet was dried and resuspended in H_2_O at 60°C for 5 min. The concentration was estimated photometrically (NanoDrop, Thermo Fisher). For cDNA synthesis, 1 μg RNA was digested with DNAse I (DNAse I, Thermo Fisher) and subsequently used for the reverse transcriptase reaction (RevertAid Reverse Transcriptase, Thermo Fisher) as described (Korn et al., 2015). The cDNA was used for assessment of the plant defense reaction using DNA primers (Supplementary Data S1) specific for different pathogenesis-related genes (PR-genes).

## Results

### Virulence Mutants *vir-49* and *vir-51* Lack a Whole Chromosome and Arrest Early in the Infection Process

**FIGURE 1:**
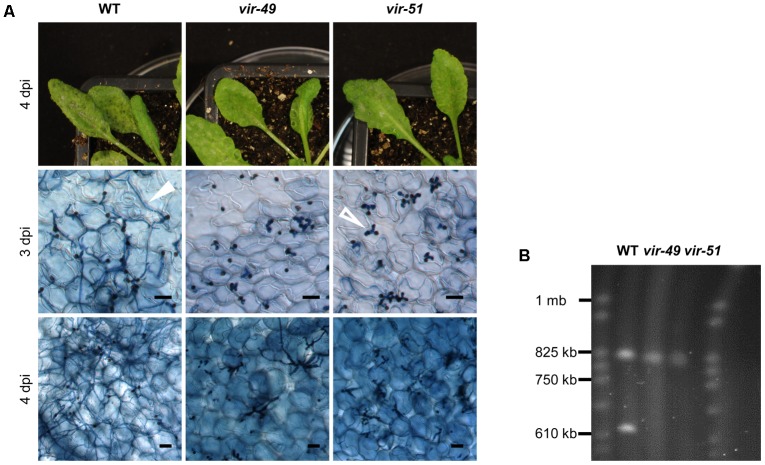
T-DNA insertion mutants *vir-49* and *vir-51* arrest early during infection. **(A)** *A. thaliana* Col-0 plants were infected with *C. higginsianum* WT and the mutant strains *vir-49* (CY5976) and *vir-51* (CY6030). The upper panel shows macroscopic symptoms at 4 dpi. The lower panels show microscopic images of trypan blue stained *A. thaliana* leaves at 3 and 4 dpi, respectively. Unfilled arrow: biotrophic primary hyphae; filled arrow: necrotrophic secondary hyphae. Scale bars = 20 μm. **(B)** Pulsed-field gel electrophoresis (PFGE) of small *C. higginsianum* chromosomes. *C. higginsianum* wild type (CY7444) and mutant conidia from strains *vir-49* and *vir-51* were analyzed on a Hoefer Hula Gel 1000 unit. Running conditions were chosen to separate chromosomes between 0.2 and 2 mb. *S. cerevisiae* chromosomes were used as a size marker (CHEF DNA Size Marker).

In a previous forward genetic screen for virulence mutants of *C. higginsianum* by insertional mutagenesis (Korn et al., 2015) we identified in total 75 mutants with diverse defects in the interaction with the model plant *A. thaliana*. Virulence mutants *vir-49* and *vir-51* showed a similar virulence phenotype characterized by their failure to effectively form necrotrophic secondary hyphae. Their ability to penetrate the *A. thaliana* host appeared normal, suggesting that they have some defect in switching from biotrophy to necrotrophic growth or fail to overcome host plant defense. Infection symptoms of Col-0 plants with these mutants (*vir-49* and *vir-51*) can be seen in **Figure 1A**. Three days post infection (3 dpi), both strains showed normal penetration rates and formed apparently normal looking bulbous primary hyphae. In contrast, the wild type (WT) already started to form necrotrophic secondary hyphae at 3 dpi. This difference was even more pronounced after 4 days. While the wild type had completely switched to necrotrophy and established a massive hyphal network in the infected host tissue, mutants *vir-49* and *vir-51* remained arrested in the stage of primary hyphae. Occasionally, some hyphae underwent a morphologic switch but developed no further (**Figure 1A**, lowest panel – *vir-49*). We additionally observed intense trypan blue staining of plant cells at spots with fungal entry, suggesting necrosis of the infected cells. Macroscopically, the mutants showed only very mild symptoms and never the strong necrotic lesions typical for the wild type. We failed to genetically link any T-DNA insertion to the observed phenotype in the two mutants by genome walker PCR (Siebert et al., 1995; Korn et al., 2015) and therefore decided to perform whole genome sequencing of the mutants to identify the mutations responsible for the phenotype. DNAs from the mutants and the parental wild type (MAFF 305635) were sequenced on an Illumina MiSeq (Supplementary Data S2). After assembling the reads to the published reference genome (Dallery et al., 2017), we found to our surprise that both mutant strains were lacking most sequence data from chromosome 11. The number of reads from the mutant DNAs that were mapped to chromosome 11 was 10-fold lower than for wild type DNA and most reads mapped to transposable elements (Supplementary Tables S1, S2). This strongly suggested that the mutant phenotype was caused by complete loss of this chromosome rather than by DNA insertions (see Supplementary Data S2 and Supplementary Table S3 for details).

We tested this further by pulsed-field gel electrophoresis (PFGE) in order to validate the absence of this particular chromosome. *C. higginsianum* harbors 12 chromosomes. While chromosomes 1–10 have sizes between 3 and 6 mb, chromosomes 11 and 12 are smaller than 1 mb (Dallery et al., 2017). Using running conditions that allow to separate small chromosomes, the two small chromosomes were detectable in the WT strain (**Figure 1B**). *Vir-49* and *vir-51*, however, were clearly lacking one of the small chromosomes with a size of approximately 620 kb. This matches the published size of chromosome 11 (646 kb) (Dallery et al., 2017). We also found that the other small chromosome was present in all three strains and showed a size of around 800 kb. In the published reference sequence of isolate IMI 349063, this small chromosome 12 only has a size of 600 kb. This also demonstrated that the MAFF 305635 isolate used for our studies contains some genomic differences to the reference isolate (Supplementary Data S2). While genome sequencing and PFGE analysis clearly demonstrated the absence of chromosome 11 in the two analyzed mutants, this did not prove that chromosomal loss is responsible for the observed virulence phenotype in *vir-49* and *vir-51*. We therefore used wild type *C. higginsianum* strains to isolate spontaneous variants that were nullisomic for chromosome 11.

### A Chromosome Loss Assay for Chromosome 11

**FIGURE 2:**
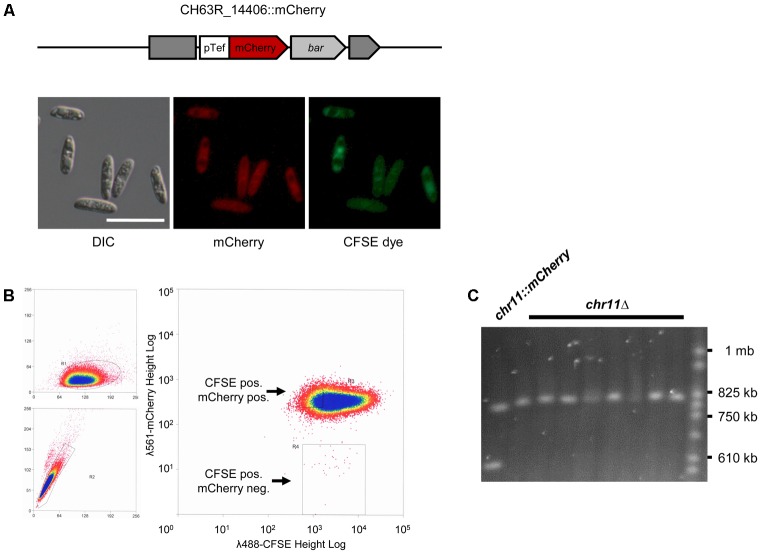
A chromosome loss assay using a targeted insertion of an mCherry gene. **(A)** Schematic representation of the CH63R_14406 locus on chromosome 11 after insertion of an mCherry reporter cassette by homologous recombination (strain CY7466). The mCherry gene was driven by a strong promotor (pTef). The *bar* gene conferring bialaphos resistance was used as a selectable marker. The dark gray blocks represent the disrupted CH63R_14406 locus. The lower images show the signals of mCherry in the conidial cytoplasm and of the fluorescent cell tracer CFSE in conidia of this strain. DIC, differential interference contrast, scale bar = 20 μm. **(B)** Flow cytometry of *chr11::mCherry* strain (CY7466) used to sort for mCherry negative conidia. We found a loss rate of 3 × 10^-4^ for chromosome 11. Left: forward and side scatter of cells. FSC-Height/ SSC-Height (top) and SSC-Height/SSC-Area (bottom). Right: fluorescent signals of CFSE at λ = 488 nm (*x*-axis) and mCherry at λ = 561 (*y*-axis). Scales are logarithmic and were adjusted to fit the range of fluorescence in the sample. The region gated for isolating mCherry neg. cells is shown as a rectangle. **(C)** Pulsed-field gel electrophoresis of chromosomes from parental *chr11::mCherry* conidia and from randomly chosen single colonies of sorted mCherry negative cells. Yeast chromosomes were used as a size marker (CHEF DNA Size Marker).

To associate the absence of chromosome 11 with the phenotype of the analyzed *vir* mutants, we established a chromosome loss assay for the generation of congenic strains with and without chromosome 11. We designed an mCherry expression cassette conferring bialaphos resistance flanked by regions of the locus CH63R_14406 located on the left arm of chromosome 11 (**Figure 2A**). Upon homologous recombination, this locus is replaced with an mCherry gene expressed from the ChTef1 promotor. A second selectable marker (nourseothricin resistance) outside the region of homology allowed to distinguish ectopic insertions from homologous recombinants after transformation of *C. higginsianum* WT (CY 7444) by *Agrobacterium tumefaciens* mediated transformation (ATMT) (Supplementary Data S1). Approximately 5 percent of the bialaphos-resistant transformants were negative for the nourseothricin marker (Supplementary Figure S1) and were also tested positive for homologous recombination by PCR on single colonies (not shown). The resulting *chr11::mCherry* strains showed strong red fluorescence in the conidial cytoplasm (**Figure 2A**).

The spontaneous loss of chromosome 11 in the tagged strain should be accompanied with the loss of mCherry fluorescence. We therefore used flow cytometry with subsequent cell sorting to isolate individual mCherry negative conidia. For gating the correct population, cells were counterstained with the green fluorescent vital stain CFSE (**Figure 2A**). FACS analysis showed that besides a large, homogenous population of double stained conidia, a very small fraction of CFSE positive, mCherry negative cells could be identified (**Figure 2B**). These were isolated by cell sorting and plated for single colonies. PCR screening showed that approximately 90 percent of mCherry negative clones had lost genes located on chromosome 11 (CH63R_14508 and CH63R_14384) (not shown). The complete loss of chromosome 11 in these isolates (*chr11*Δ) was validated by PFGE for randomly picked colonies (**Figure 2C**) and by PCR analysis of five different loci separated by approximately 100 kb on chromosome 11 (Supplementary Figure S1). Interestingly, loss of chromosome 11 could be detected without extensive subculturing of the original *chr11::mCherry* strains.

### Chromosome 11 of *C. higginsianum* Is Essential for Virulence on *A. thaliana* but Does Not Affect Vegetative Growth

**FIGURE 3:**
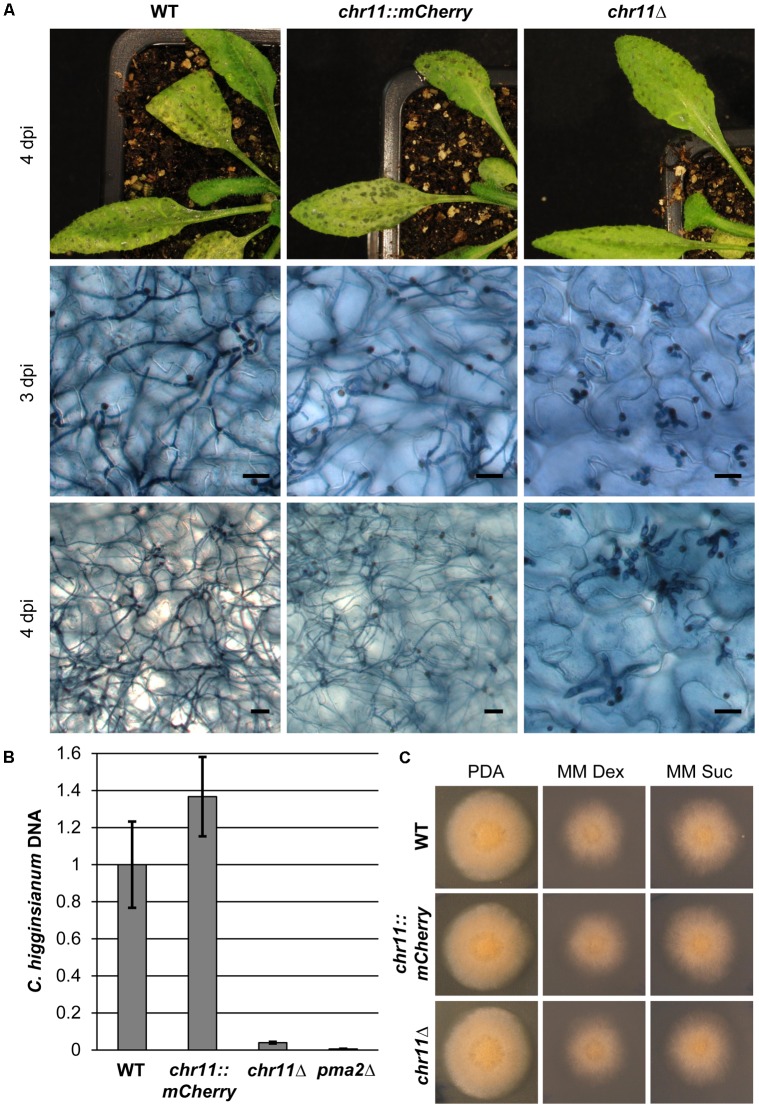
Strains lacking chromosome 11 arrest in the post penetration stage of infection. **(A)** *A. thaliana* Col-0 plants were infected with *C. higginsianum* WT, a strain lacking chromosome 11 (CY7548) (*chr11*Δ) and the respective mCherry tagged parental strain (CY7466) (*chr11::mCherry*). The upper panel shows macroscopic symptoms at 4 dpi. The two lower panels show microscopic images of trypan blue stained *A. thaliana* leaves at 3 and 4 dpi, respectively. Scale bars = 20 μm. **(B)** Relative amount of fungal DNA from infected host tissue at 4 dpi. A *pma2*Δ mutant strain (CY6153) which only forms appressoria and does not penetrate the plant was used as additional control. DNA amount was assessed via qPCR, corrected for leaf area and normalized to WT infection. Samples of five biological replicates per strain were taken after 90 h. Error bars show the standard error for each data set. **(C)** Vegetative growth of the respective strains on potato dextrose agar (PDA) and minimal media with dextrose (MM Dex) or sucrose (MM Suc).

To validate the connection between loss of chromosome 11 and loss of virulence, we infected *A. thaliana* Col-0 plants with variants that have lost chromosome 11 (*chr11*Δ), the corresponding parental strain *chr11::mCherry* and *C. higginsianum* wild type (**Figure 3A**). There were no obvious differences between infections with either the wild type or the tagged *chr11::mCherry* strain. At 3 dpi both strains had switched to necrotrophic growth. At 4 dpi, the infected leaves were abounded with secondary hyphae and the plants showed strong macroscopic symptoms. The *chr11*Δ variant, however, showed a defect closely resembling the virulence phenotype originally observed for *vir-49* and *vir-51* mutants (**Figure 3A**). Three days after infection the fungus had penetrated the plant cells and established normal looking primary hyphae but showed no visible switch in hyphal morphology. Four days post infection almost all hyphae remained stuck as large bulbous hyphae. Only few (<3%) primary hyphae of the *chr11*Δ strain produced filamentous secondary hyphae. Identical results were found for several independently isolated *chr11*Δ strains (see below and not shown).

We performed quantitative PCR on fungal genomic DNA of infected leaves approximately 4 days post infection to quantify fungal proliferation *in planta* (**Figure 3B**). As expected for an aborted infection, leaves infected with *chr11*Δ showed 25 times less fungal DNA than leaves infected with the wild type. In order to further evaluate the limited proliferation of *chr11*Δ cells, we compared it to a penetration defective *C. higginsianum* mutant lacking a virulence specific proton pump (*pma2*Δ) that replicates twice during appressoria formation but never enters the host cell (Korn et al., 2015). The *chr11*Δ mutant generated approximately 6.5 times more DNA during infection compared to *pma2*Δ. The wild type, however, generated approximately 160 times more DNA in the host tissue than the penetration mutant (**Figure 3B**). In summary, our data clearly demonstrate that infection was aborted early during infection after few replication cycles in the absence of chromosome 11.

To more carefully investigate if the *chr11*Δ strains show additional phenotypes we analyzed the formation of appressoria *in vitro*, i.e., on petri dishes and *in planta* but did not see any difference between *chr11*Δ and the WT *C. higginsianum* strain. Importantly, the number of appressoria that successfully penetrated the plant cells during infection was unaltered in the *chr11*Δ mutants (Supplementary Figure S2). In addition, we noticed some atypical lesions on leaves 4 days after infection which stained heavily with trypan blue which may indicate host cell necrosis, i.e., defense responses (Supplementary Figure S3). Such areas were absent in WT infections. Moreover, we observed that the staining of large, bulbous hyphae of *chr11*Δ strains at 4 dpi was less intense and the hyphae were more vacuolated compared to the bulbous hyphae after 3 days (Supplementary Figure S3B). This may suggest that the hyphae are no longer viable at this point of the infection.

Finally, the loss of chromosome 11 had no visible effect on the vegetative fitness or colony morphology on rich or minimal media (**Figure 3C**). The functions of genes on chromosome 11 therefore appear to be confined to specific roles during the infection process.

### Altered Host Response in the Absence of Chromosome 11

**FIGURE 4:**
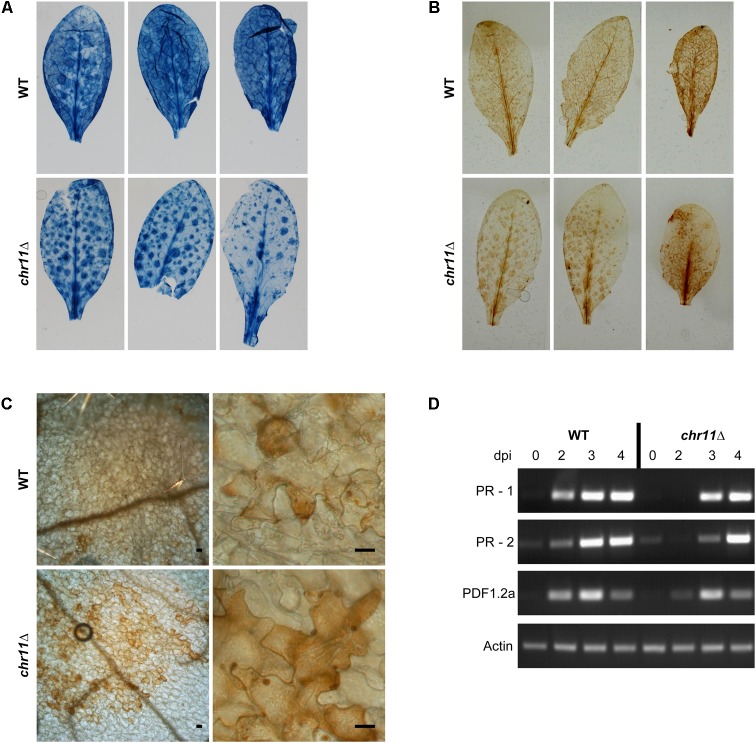
Plant response to *C. higginsianum* lacking chromosome 11. **(A)** *A. thaliana* Col-0 plants were infected with *C. higginsianum* WT (CY7444) and *chr11*Δ mutant (CY7548). One representative leaf each from 3 infected plants per strain were harvested at 4 dpi and stained with trypan blue to stain fungal cells and dead plant tissue. After destaining, macroscopic photos were taken. **(B)** Macroscopic images of 3,3′-diaminobenzidine (DAB) stained leaves at 4 dpi for visualization of reactive oxygen species (ROS). **(C)** Microscopic images of representative DAB stained leaves at 4 dpi. Scale bars = 20 μm. **(D)** RT-PCR of plant PR genes at different time points during the infection of *chr11*Δ and the *C. higginsianum* wild type.

We observed atypical lesions on *A. thaliana* leaves infected with *chr11*Δ strains. These areas were macroscopically visible in stained samples (**Figure 4A**) and distributed on the whole leaf beneath spots with signs of fungal infection. As these areas contained very little fungal material, they most probably correspond to dead *Arabidopsis* cells which also get stained with trypan blue (Keogh et al., 1980; Pogany et al., 2009). The lesions are reminiscent of a hypersensitive response of *A. thaliana* which may fail to be suppressed by the pathogen when genes from chromosome 11 are missing.

To characterize this further, we performed ROS staining using 3,3′-diaminobenzidine (**Figures 4B,C**). At 4 dpi, we found the macroscopic DAB staining of leaves infected with *chr11*Δ to show the same pattern as the staining of dead plant cells with trypan blue. Microscopically, we observed clusters of cells with stronger DAB staining in the *chr11*Δ infection which could be assigned to the macroscopically visible areas. In contrast, leaves infected with *C. higginsianum* wild type were already fully colonized by the fungus at this time point with many dead plant cells and less DAB staining. The observed and altered host response against *chr11*Δ suggested that the inability of *chr11*Δ mutants to switch to necrotic growth is due to successful plant defense rather than a consequence of a defective fungal regulation. We also analyzed the expression of plant pathogenesis-related genes by RT-PCR at different time points during the infection with *chr11*Δ and the *C. higginsianum* wild type (**Figure 4D**). Both fungal strains induced expression of the salicylic acid induced genes PR-1 and PR-2 (Penninckx et al., 1996). Also the plant defensin PDF1.2a which is induced by the jasmonic acid pathway (Uknes et al., 1992) was induced by both strains. However, the three genes appear to be induced earlier in the wild type infection.

### Plant Tryptophan P450 Monooxygenases cyp79b2 and cyp79b3 Are Required to Prevent Infection by *C. higginsianum* Mutants Lacking Chromosome 11

Having observed an altered host reaction against strains lacking chromosome 11 and *C. higginsianum* wild type, we tested a collection of *A. thaliana* mutants with defects in different layers of plant defense. If the phenotype of *chr11*Δ strains were due to the inability to suppress plant defense, we hypothesized that such strains should regain virulence in the absence of critical plant defense pathways.

**FIGURE 5:**
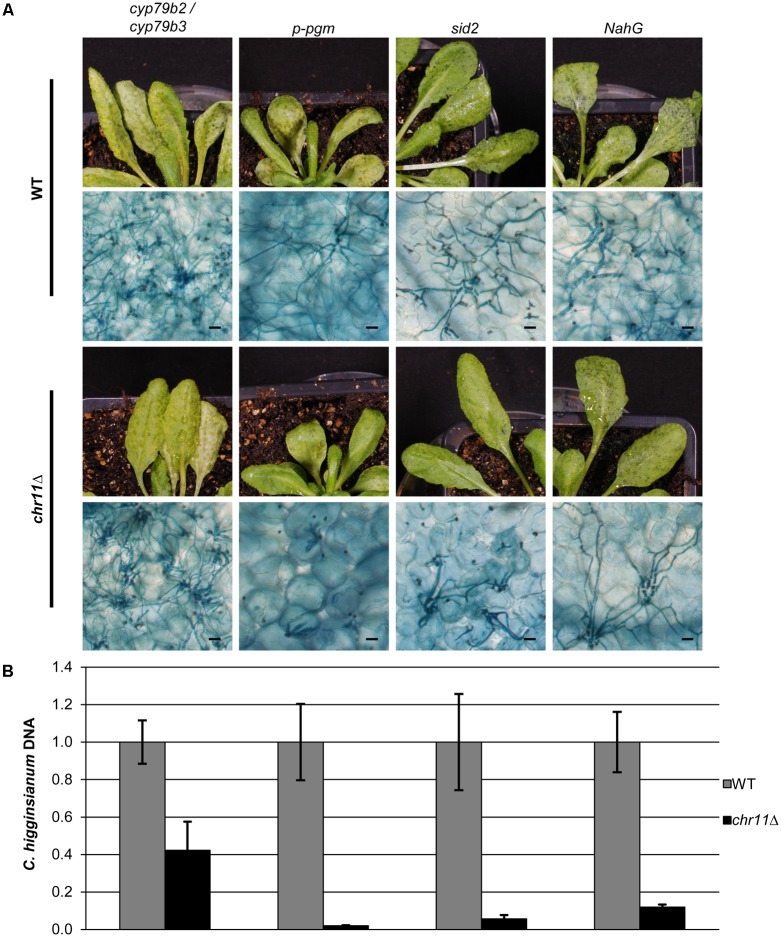
Chromosome 11 is required to suppress post penetration plant defense. **(A)** Macroscopic and microscopic symptoms of WT (upper panel) and *chr11*Δ (lower panel) infection of the analyzed mutant lines (*p-pgm*, *sid2*, *NahG, cyp79b2/cyp79b3*) after 90 hpi. Scale bars = 20 μm. **(B)** *C. higginsianum* WT and *chr11*Δ strains were used to infect different *A. thaliana* mutants. Relative amount of fungal DNA from infected *A. thaliana* lines (*p-pgm*, *sid2*, *NahG, cyp79b2/cyp79b3)* was analyzed at 90 hpi. DNA amount was assessed via qPCR, corrected for leaf area and normalized to *Ch* WT infection of each mutant line. Samples of five biological replicates per strain were taken. Error bars show the standard error for each data set.

We therefore infected the *A. thaliana* double mutant *cyp79b2/cyp79b3* (**Figure 5**). This hypersusceptible mutant cannot convert tryptophan to indole-3-acetaldoxime (IAOX) and is therefore lacking a number of defense related metabolic products like indole glucosinolates and camalexin. This mutant is highly susceptible to various fungal pathogens including *Colletotrichum* species (Bednarek et al., 2009; Sanchez-Vallet et al., 2010; Hiruma et al., 2013). Already 3 days post infection the *C. higginsianum* WT strain produced necrotrophic hyphae in large areas. Four days post infection, the tissue was massively colonized by the WT and acervuli were detected. The *chr11*Δ strain not only established primary hyphae after 3 days, but at 4 dpi the strain also colonized large parts of the host tissue with necrotrophic secondary hyphae, indicating that *chr11*Δ strains can be virulent on *A. thaliana* when plant defense is impaired (**Figure 5A** and Supplementary Figure S4).

To exclude that the colonization of *A. thaliana cyp79b2/cyp79b3* double mutants by *chr11*Δ was due to pleiotropic defects of the plants, we tested additional *A. thaliana* mutants and quantified the virulence of *C. higginsianum* wild type and the *chr11*Δ strain via quantitative PCR. Infections of transgenic *NahG* plants, which do not accumulate salicylic acid (Delaney et al., 1994), *sid2* plants which are defective for salicylic acid signaling and infections of the starch free *p-pgm* mutant which is more susceptible toward *C. higginsianum* were compared with *cyp79b2/cyp79b3* mutant plants (**Figure 5**). While the *chr11*Δ strain colonized the *cyp79b2/cyp79b3* successfully and generated more than 40% of the fungal DNA observed in infections with *C. higginsianum* wild type, the *chr11*Δ strain was unable to establish secondary hyphae on the *p-pgm* mutant and generated only approx. 2% of the fungal biomass observed for the *C. higginsianum* wild type (**Figure 5B**). Given the fact that the *p-pgm* line is generally more susceptible to *C. higginsianum* but refractory to infection with *chr11*Δ, this strongly suggests that the colonization of *cyp79b2 / cyp79b3* double mutants by *chr11*Δ is due to a specific defense defect and not due to pleiotropic effects. On *sid2* mutants and the *NahG* lines the *C. higginsianum* strain lacking chromosome 11 showed very limited proliferation compared to WT *C. higginsianum* (6–12% of WT). Consistent with that, only a few *chr11*Δ primary hyphae switched to necrotrophic hyphae and their growth remained locally restricted (**Figure 5A**). This may indicate that genes on chromosome 11 also effect SA-mediated defense.

We also analyzed additional defense mutants (Supplementary Figure S4). The *cerk1-2* mutant, defective for a pattern recognition receptor (PRR) sensing chitin (Miya et al., 2007), showed no increased susceptibility when infected with the *chr11*Δ strain. The same was observed for the preinvasion defense defective mutant *pen2-2* (Bednarek et al., 2009). In addition, we also analyzed the camalexin defective *pad3* mutant (Narusaka et al., 2004) but did not observe significant colonization by the *chr11*Δ strain.

### Chromosome 12 Is Not Required for Virulence on *A. thaliana*

**FIGURE 6:**
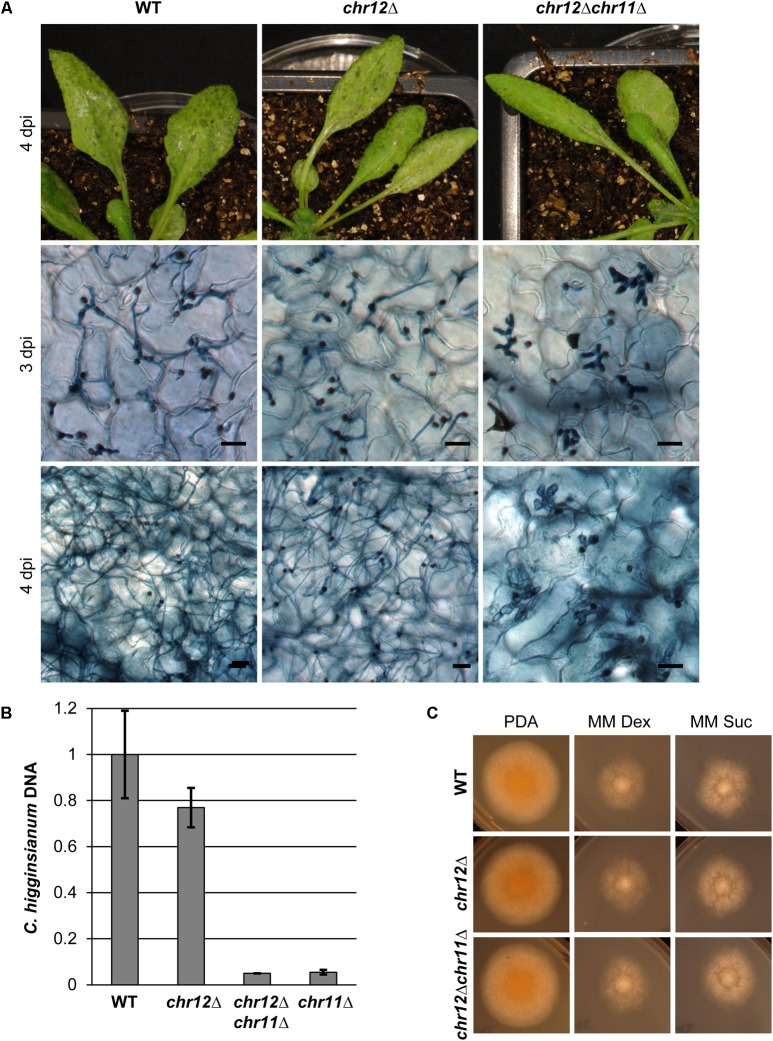
Chromosome 12 is not required for virulence on *A. thaliana*. **(A)** *A. thaliana* Col-0 plants were infected with *C. higginsianum* WT, a strain lacking chromosome 12 (CY7554) (*chr12*Δ) and a strain lacking both small chromosomes (CY7589) (*chr12Δchr11*Δ). The upper panel shows macroscopic symptoms at 4 dpi. The two lower panels show microscopic images of trypan blue stained *A. thaliana* leaves at 3 and 4 dpi, respectively. Scale bars = 20 μm. **(B)** Relative amount of fungal DNA from infected host tissue at 4 dpi. The DNA amount was assessed via qPCR, corrected for leaf area and normalized to WT infection. Samples of four biological replicates per strain were taken at 4 dpi. Error bars show the standard error for each data set. **(C)** Vegetative growth of the respective strains on potato dextrose agar (PDA) and minimal media with dextrose or sucrose.

*Colletotrichum higginsianum*’s two small chromosomes 11 and 12 have similar properties. Both are very small, harbor many transposable elements and encode potential effector genes (Dallery et al., 2017). We therefore also generated strains that had spontaneously lost chromosome 12, using the chromosome loss assay described above but with an mCherry gene integrated at the CH63R_14534 locus from chromosome 12 (**Figure 6** and Supplementary Figure S5). Similar to the *chr11*Δ strains, *chr12*Δ strains were readily obtained and were not impaired in growth on synthetic media (**Figure 6C**).

In contrast to *chr11*Δ strains, however, we did not detect any obvious differences in virulence between *C. higginsianum* wild type and the chromosome 12 loss mutant *chr12*Δ (**Figure 6A**). Both established biotrophic primary hyphae in the host tissue at 3 dpi and showed a morphological switch to necrotrophy in some areas. Four days after infection, leaves showed heavy macroscopic symptoms in both cases. Microscopically, colonization with necrotrophic secondary hyphae was similar for both strains.

We also generated a double mutant lacking both small chromosomes by using *chr12*Δ as the parental strain for the tagging of chromosome 11 and for the subsequent isolation of mCherry negative variants lacking both chromosomes (Supplementary Figure S5). Again, such *chr12Δchr11*Δ strains were easily obtained by cell sorting and their growth on synthetic media was comparable to WT (**Figure 6C**). In virulence assays, the phenotype of the double mutant resembled the virulence defects of the *chr11*Δ in all regards (**Figure 6A**), suggesting that the additional loss of genes on chromosome 12 does not enhance the virulence defect of *C. higginsianum* lacking chromosome 11. We also performed quantitative PCR on fungal genomic DNA of infected leaves 4 days post infection to assess the fungal proliferation *in planta* (**Figure 6B**). As observed in microscopy, there was no significant difference between the wild type and the *chr12*Δ mutant and no enhanced phenotype in the double mutant (*chr12*Δ*chr11*Δ) compared to *chr11*Δ.

### Differences Between *C. higginsianum* Strains

As the karyotype of the MAFF 305635 strain showed differences to strain IMI 349063 regarding the size of chromosomes 11 and 12 (see above), we compared the entire genome sequences by mapping MiSeq illumina reads of the MAFF 305635 WT strain to the reference genome of IMI 349063 (Supplementary Data S2). The PFGE analysis (**Figure 1** and data not shown) of the MAFF 305635 isolate showed that chromosome 12 was ∼200 kb larger than reported for the reference strain, while chromosome 11 lacked roughly 30–50 kb relative to the reference sequence from IMI 349063. The sequence alignment to the reference confirmed this difference as no reads from the MAFF 305635 strain mapped to annotated genes in the region from position 355,533 to position 406,259 corresponding to a 50 kb region between gene CH63R_14468 and gene CH63R_14477 of the reference genome (Supplementary Data S2). Some small additional regions in which the two strains differ were identified by looking for segments of low coverage in the MAFF 305635 sequencing (Supplementary Tables S2, S3 and Supplementary Data S2). This analysis identified genes potentially lacking in MAFF 305635, while the absence of a complete genome assembly for the MAFF 305635 strain did not allow to identify genes specific to MAFF 305635.

In order to evaluate how divergent the two *C. higginsianum* isolates are, we analyzed differences in the coding regions between the reference assembly and the MAFF 305635 isolate using the variant caller SnpEfff (Supplementary Data S2 and Supplementary Table S4). Most of the genome was very similar to the reference and showed about 1 SNP per 1 kb of coding region (**Table 1** and Supplementary Table S4). This variation is very similar to the variations found between pairs different isolates of the human fungal pathogen *Aspergillus fumigatus* (1 SNP/kb coding) (Takahashi-Nakaguchi et al., 2015).

**Table 1:** Sequence variations in coding regions of MAFF 305635 and IMI 349063.

Chromosome	Size of coding regions [kb]^a^	Coding variants (SNPs)	Coding variants/ Size [kb]
1	2551	2016	0.79
2	2506	1874	0.75
3	2619	3549	1.36
4	2213	1731	0.78
5	2112	1401	0.66
6	1768	1946	1.10
7^b^	1816	2426	1.34
8	1742	2408	1.38
9	1635	1502	0.92
10	1381	1117	0.81
11	141	17	0.12
12	128	677	5.27
Sum (1–12)	20612	20664	1.00


*^*a*^Numbers are from reference assembly. ^*b*^LTAN01000007.*

Two large regions in the genome showed notable differences to this picture. One region corresponds to chromosome 11 and was more conserved between IMI 349063 and MAFF 305635 with a total of only 17 SNPs in all coding regions between the strains (0.12 SNPs/kb). The other region corresponds to chromosome 12 which showed approximately 5 SNPs per 1 kb coding region. This may indicate that chromosome 11 is more conserved than the rest of the genome and may have only recently been acquired, while genes on chromosome 12 may have undergone significant, possibly, strain specific changes in the two isolates.

## Discussion

Dispensable chromosomes have been described in diverse organisms. In some cases, in particular in sexual species, it has been argued that they may selectively provide advantages and/or adaptation to special conditions (Jones, 1991). In fungi, dispensable chromosomes have been reported in several species, in particular in *Fusarium solani* (Miao et al., 1991). Later lineage-specific (LS) chromosomes were found in other pathogenic fungi, for instance *Alternaria alternata* (Hatta et al., 2002) or in *Fusarium oxysporum* (Ma et al., 2010; Vlaardingerbroek et al., 2016a,b). Dispensable chromosomes often differ from the core chromosomes of the particular organisms in their size, gene content or sequence characteristics (Moller and Stukenbrock, 2017). In *Colletotrichum* species dispensable chromosomes have been noticed in C. *gloeosporioides* (Masel et al., 1996) and it was reported that *C. higginsianum* and *C. graminicola* harbor small mini chromosomes (Taga et al., 2015; Dallery et al., 2017) but no roles in virulence have been reported for these small chromosomes.

We here show that the loss of a dispensable chromosome in *C. higginsianum* has severe consequences on its ability to cause disease while having no effects on vegetative growth. The presence of this chromosome seems to provide a specific advantage for *C. higginsianum* as a pathogen. In particular, it enables the fungus to suppress post invasion plant defense mechanisms. We also found that a second dispensable mini chromosome can be lost during saprophytic growth without any effect on growth or pathogenicity. The two dispensable chromosomes are much smaller than the other 10 chromosomes which range from 3 to 6 mb (O’Connell et al., 2012). Chromosomes 11 and 12 account for only 3% of the genome, covering approximately 1% of annotated genes but are enriched for genes encoding putative secreted effector proteins (Dallery et al., 2017).

### How Often Does *C. higginsianum* Lose Mini Chromosomes?

Our chromosome loss assay was based on the spontaneous loss of tagged chromosomes 11 or 12, respectively, during mitotic division and growth on agar plates. Vegetatively grown cultures of *chr11::mCherry* and *chr12::mCherry* produce conidia lacking the respective chromosome at a frequency of more than 1 × 10^-4^. This rate is remarkably high compared to the numbers reported for *Fusarium* where the loss rate of a lineage specific LS chromosome using a similar experimental approach was approximately 3 × 10^-5^ (Ma et al., 2010; Vlaardingerbroek et al., 2016b). This frequency may be directly related to chromosome size as chromosomes 11 and 12 from *C. higginsianum* are much smaller than the LS chromosomes of *Fusarium*. Chromosome loss can only be observed and studied when the respective chromosome is not essential or important for growth. Therefore, documented frequencies of chromosome loss usually are rare. But there are numbers for mitotic stability of centromeric plasmids and chromosomes in yeast. Yeast plasmids with replication origins and centromeres are lost at very high frequencies of up to 1 × 10^-2^. For linear yeast chromosomes, the segregation frequencies appear to be size dependent with artificial mini chromosomes (less than 200 kb) being lost at a rate of about 1 × 10^-3^ while larger chromosomes (e.g., ChrVII, 1091 kb) are 100 times more stable (Hartwell et al., 1982; Hieter et al., 1985). In light of these numbers, a specific active mechanism may not be required to explain the high frequency of mini chromosome loss in *C. higginsianum*. This may also explain why we found loss of chromosome 11 in two independently isolated *vir* mutants. The high spontaneous rate of chromosome loss for *C. higginsianum* mini chromosomes further suggests that chromosome loss was not dependent on the transformation by ATMT during the original insertional mutagenesis (Korn et al., 2015). Chromosomes that are not essential can be lost by three possible mechanisms when checkpoint mechanisms are overridden. First, a late replicating chromosome with few origins of replication may become left behind. Second, a failure in chromosome cohesion during mitosis might lead to non-disjunction. Finally, meiotic cohesion problems may lead to chromosome loss. All of these mechanisms may be more error prone when chromosomes are small, i.e., have fewer replication origins (ARS sequences) and fewer sites of cohesion or meiotic chiasmata. Error prone non-disjunction may further be enhanced in systems where the number of microtubules is limiting as was suggested for yeast (Hieter et al., 1985). In fact, yeast artificial chromosomes with sizes close to chromosomes 11 and 12 from *C. higginsianum* have been reported to be unstable. It is, however, also quite possible that the rate of chromosome loss we observed is neither specific to mini chromosomes nor a selected feature of *C. higginsianum* but occurs in all systems, yet without being noticed as the resulting cells may simply die or may have no obvious phenotype.

### Why Should Chromosomes 11 and 12 Be Mitotically Instable?

If the loss of mini chromosomes were harmful one might expect such small chromosomes to fuse with others in order to gain stability. It is therefore tempting to speculate that, rather than being a problem, their instability is a feature that is selected for by small chromosome size. Chromosome loss may be the simplest mechanism to escape those host specific defense mechanisms that depend on the presence of corresponding avirulence genes in the pathogen. Loss of function mutations in pathogen effector genes have indeed been shown to be a key mechanism to escape plant defense (Jones and Dangl, 2006). Since *C. higginsianum* belongs to fungi that reproduce mostly asexual, the loss of mini chromosomes may be a dead end unless they are regained by cell fusion, conidial anastomosis or other mechanisms (Seidl and Thomma, 2014). Indeed, such events have been described in the genus of *Colletotrichum*. Conidial anastomosis and parasexual recombination was shown for *Colletotrichum lindemuthianum* (Ishikawa et al., 2012) and the transfer of supernumerary chromosomes between two biotypes was observed in *Colletotrichum gloeosporioides* (He et al., 1998). The loss and parasexual gain of dispensable mini chromosomes may be an alternative resource of expanding the genetic repertoire quite similar to bacterial episomal plasmids. Such recent gain of extra chromosomes may be a means for the fast adaptation to changing environmental situations and for the adaptation to changing hosts and hosts evolving novel resistance mechanisms.

### Sequence Variations in Chromosomes 11 and 12

The evolutionary history of individual genomic regions is thought to be reflected in coding sequence variations as well as genomic and genetic rearrangements (Dong et al., 2015; Moller and Stukenbrock, 2017). Most of the core genome of *C. higginsianum* shows a variation rate in coding regions of 1 SNP/kb between the two different *C. higgisianum* isolates analyzed. This is similar to the variation seen when different *Verticillium dahliae* strains were compared (de Jonge et al., 2013; Faino et al., 2016) and comparable to the situation for different genetically isolated yeast isolates (Peter et al., 2018). In contrast, the conservation between coding regions on chromosome 11 is well above average (**Table 1**), suggesting that the encoded genes are subject to purifying selection. It is striking that genes on chromosome 11 show such little variability, since the two compared isolates originate from very different locations. The reference isolate IMI 349063 was isolated in Trinidad and Tobago while MAFF 305635 used here originates from Japan. For genes on chromosome 12 the situation is exactly the opposite, significant variation in their coding regions is observed and is 5 times higher than for the rest of the genome (**Table 1**). This may indicate that chromosome 12 has undergone recent changes, possibly reflecting some adaptation to the different environments. We also noticed that according to the data reported for gene expression in planta (Dallery et al., 2017) genes from chromosome 12 contribute only very little to fungal gene expression during infection of *A. thaliana*. It is also noteworthy that chromosome 11 may be variable with regard to gene number because a region of 50 kb from the reference appeared to be absent from the isolate used in this study. This region encompasses nine genes (CH63R_14468 to CH63R_14477) including five genes annotated as hypothetical protein and two genes encoding C14 peptidases.

### Loss of Chromosome 11 Leads to Increased Success of Post Penetration Resistance

Our results clearly showed that the loss of chromosome 11 leads to a specific defect in suppression of plant host response mechanisms, because typical signs of necrotrophic colonization are restored when tryptophan derived secondary metabolites are missing. We excluded any indirect pleiotropic effect by showing that starch free *A. thaliana p-pgm* plants in the *Ler-0* background which are much more susceptible to *C. higginsianum* infection (Engelsdorf et al., 2013) remain fully resistant to *chr11*Δ strains.

Which specific plant defense mechanisms are no longer suppressed when chromosome 11 is missing? *A. thaliana* uses different lines of defense to provide resistance toward hemibiotrophic fungi. Most important are: Salicylic acid (SA) and Jasmonic acid (JA) induced by PAMP (pathogen-associated molecular pattern) recognition (Narusaka et al., 2004; Liu et al., 2007), the phytoalexin camalexin (Narusaka et al., 2004) and tryptophan derived secondary metabolites including indole glucosinolates (Hiruma et al., 2013).

We analyzed a subset of these pathways by infecting the respective *Arabidopsis* defense mutants. We found no effect on the ability of *chr11*Δ to colonize *Arabidopsis* when the chitin PAMP receptor CERK1 is missing, which fits to the finding that CERK1 plays a minor role in defense against *C. higginsianum* (Miya et al., 2007). Most likely, other chitin receptors or other PAMPs are more important for *C. higginsianum* detection. However, the existence of chitin masking LysM domain proteins in *C. higginsianum* suggests that chitin is important (Takahara et al., 2016; Dallery et al., 2017). In addition, chitin deacetylation and dynamic changes in cell wall components may be important to hamper chitin detection (Sanchez-Vallet et al., 2015) as it is known for *Magnaporthe* (Fujikawa et al., 2012). Both SA and JA are known to be upregulated upon infection with pathogens as a part of the innate immune system and to trigger a specific set of pathogenesis-related (PR) genes (Thomma et al., 1998). Infection with *C. higginsianum* leads to induction of PR transcripts, like SA-dependent PR1 and PR2 while PDF1.2 is induced by JA-dependent signaling (Narusaka et al., 2004; Liu et al., 2007). We found that ectopic *NahG* expression and to a lesser extent *sid2* mutations which both result in reduced SA levels, allow locally limited colonization by *chr11*Δ strains suggesting that genes on chromosome 11 may also interfere with SA-mediated defense responses. For *Pseudomonas syringae* and the oomycete *Peronospora parasitica*, it was reported that *NahG* mutants affect susceptibility more strongly than the *sid2* mutant (Nawrath and Métraux, 1999) which fits our observations. However, the spreading of the *chr11*Δ strain was very limited and not comparable to the *C. higginsianum* WT (**Figure 5**). The double mutant *cyp79b2/cyp79b3*, defective for two monooxygenases was the only *Arabidopsis* line susceptible to *chr11*Δ. We therefore assume any of the several metabolites downstream of their product indole-3-acetaldoxime (IAOx) to be a key component in the defense against *C. higginsianum*. IAOx is the starting component of several biochemical pathways including auxin, camalexin and indole glucosinolates. Although camalexin was reported to play an important role in the defense against *C. higginsianum* (Narusaka et al., 2004), mutants defective for this compound (*pad3*) were not more susceptible to the *chr11*Δ strain. The same was true for *pen2-2*, a preinvasion defense defective mutant involved in indole glucosinolate biosynthesis (Bednarek et al., 2009). Either IAOx synthesis itself or other tryptophan derived secondary metabolites must therefore be targeted by genes on chromosome 11 to successfully suppress infection by *C. higginsianum*. Our fortuitous finding of strains lacking chromosome 11 should now allow to identify the relevant genes.

### Potential Pathogenicity Genes on Chromosome 11

The genome sequencing of *C. higginsianum* (Dallery et al., 2017) revealed shared characteristics for chromosome 11 and 12 which differ from the core genome. Both display a lower gene density, are significantly enriched with transposable elements and have a higher AT-content. Most interestingly, both are also enriched in genes encoding potential effector proteins and proteins of unknown function. We did not observe any virulence defects in *chr12*Δ strains, suggesting no contribution in the pathogenicity against *A. thaliana*. In contrast, we found chromosome 11 to be directly associated with virulence, in particular in the suppression of post invasion defense mechanisms, indicating that a certain subset of genes from chromosome 11 have critical functions in manipulating plant host responses. In total, there are 7 candidate effector genes (ChEC) on chromosome 11 which may contribute to fungal virulence. ChEC7 is duplicated on both arms of the chromosome. This duplicated region harbors three genes within 7 kb all of which have homologs in other species. ChEC12 and ChEC12a exhibit high similarity. Both genes are expressed during the biotrophic phase. ChEC21 and ChEC24 are not species specific genes and are expressed during the initial phases of infection. Interestingly, ChEC116 is expressed during the whole infection and is one of the most highly expressed genes in the biotrophic phase of *C. higginsianum* (Dallery et al., 2017).

Moreover, there are in total five genes annotated as Nudix domain-containing proteins which code for hydrolases of nucleoside diphosphate linked compounds and which have been suggested to have important function in plant pathogens (Dong and Wang, 2016). A Nudix domain-containing protein has been reported to be involved in the switch from biotrophy to necrotrophy in *C. truncatum* (Bhadauria et al., 2013).

In summary, chromosome 11 harbors a number of potential genes which can now be selectively analyzed to evaluate their possible function in suppressing post invasion host defense.

## Data Availability Statement

The genome data for this study can be found in the sequence read archive (SRA) at NCBI (accession number of bioproject: PRJNA427316 [https://www.ncbi.nlm.nih.gov/bioproject/PRJNA427316]).

## Author Contributions

CK conceived the project. P-LP, MD, and JS performed the experiments. CK, P-LP, and MD analyzed the experimental data. LT and CK performed the bioinformatic analysis. P-LP and CK wrote the manuscript. All authors edited the manuscript.

## Conflict of Interest Statement

The authors declare that the research was conducted in the absence of any commercial or financial relationships that could be construed as a potential conflict of interest.

## Footnotes

1 http://www.bioinformatics.babraham.ac.uk/projects/fastqc/

2 https://cutadapt.readthedocs.io/
