# Supplementary material for: A Dispensable Chromosome Is Required for Virulence in the Hemibiotrophic Plant Pathogen Colletotrichum higginsianum
Source: Front Microbiol. 2018 May 18;9:1005. doi: 10.3389/fmicb.2018.01005 (PMC5968395; doi:10.3389/fmicb.2018.01005)
Supplement: Supplementary file 2 [file Data_Sheet_1.PDF]

Supplementary data 1

Oligonucleotides used, plasmids, additional strains and plasmid constructions

Oligonucleotides

Name	Sequence 5' --> 3'	Description
Ch TrpC fw	AAGTTCAGACTGCCGAAG	*ChTrpC-α* qPCR primer
Ch TrpC rev	TCAGCCTGCTTGTTGTGT	*ChTrpC-α* qPCR primer
CK2668	GCCCTATTCTCGCTCGTCTTCC	*ChTUBULIN-α* RT-PCR primer
CK2669	GGGCTCCAAATCGCAGTAAATG	*ChTUBULIN-α* RT-PCR primer
CK3831	ATGAGCCCAGAACGACGC	bar-specific primer for verification of in locus integration of bialaphos resistance gene
CK4042	ATCTCGAACTCGTGGCCGTTCA	mCherry-specific primer for verification of in locus integration
CK5192	CGTTTCTGACGGGTTGCTTCA	primer for amplification of CH63R_14508 on chromosome 11
CK5193	CAAGGGGGAGGAAAGGTTGAAT	primer for amplification of CH63R_14508 on chromosome 11
CK5196	TGCCGACAATGCTTTCGTAGA	primer for amplification of a fragment of CH63R_14534 on chromosome 12
CK5197	TCAAGTCGTGGCAAAGTTCCT	primer for amplification of a fragment of CH63R_14534 on chromosome 12
CK5207	GGGGACAGCTTTCTTGTACAAAGTGGAA CACCGCAAAGGGTTCACGGCAGAT	primer for amplification of a fragment of CH63R_14500 on chromosome 11
CK5208	GGGGACTGCTTTTTTGTACAAACTTGTC GACGCAAGAGGGAAAAGGCACGA	primer for amplification of a fragment of CH63R_14500 on chromosome 11
CK5209	GGGGACAACTTTGTATAGAAAAGTTGTT AGCCCCGCTGCCTACTTGTTCCTT	primer for amplification of a fragment of CH63R_14381 on chromosome 11

CK5213	GGGGACAACTTTGTATAGAAAAGTTGTTTTCCAACCCACATTCCAGGGGCGA	primer for amplification of a fragment of CH63R_14517 on chromosome 11
CK5214	GGGGACAACTTTGTATAATAAAGTTGTGCCGTTCTCACCCTCCTCGCATCT	primer for amplification of a fragment of CH63R_14517 on chromosome 11
CK5230	GGGGACAGCTTTCTTGTACAAAGTGGAATCCGAAGAACAGGCATTGTTAG	primer for amplification of a fragment of CH63R_14384 on chromosome 11
CK5231	GGGGACTGCTTTTTTGTACAAACTTGTTGTTAGTTTGGCGTTCATTAGCC	primer for amplification of a fragment of CH63R_14384 on chromosome 11
CK5236	GGGGACAACTTTGTATAGAAAAGTTGTTTGAGCGGAGTGGCGAGAGGAAT	primer for amplification of a fragment of CH63R_14428 on chromosome 11
CK5237	GGGGACAACTTTGTATAATAAAGTTGTGGAAGCTAAGTCCTTGTTTGCA	primer for amplification of a fragment of CH63R_14428 on chromosome 11
CK5267	CTTGTACAAAGTGGAAGTTTAAACTCTTGTGGCCTTCGCAATC	primer for amplification of 5' homology region of CH63R_14406
CK5268	CTGCATGGTCGTTTAAACACGCGATGCTGAGTTGGAAATG	primer for amplification of 5' homology region of CH63R_14406
CK5269	AGCATCGCGTGTTTAAACGACCATGCAGACATACCTAG	primer for amplification of promotor region of CH63R_07477 with corresponding ends for chromosome 11 homology at CH63R_14406
CK5270	TCCTCGCCCTTGCTCACCATTTTGGCGGTTCTGGATCG	primer for amplification of the promotor region of CH63R_07477
CK5271	CTTGTACAAAGTGGAAGTTTAAACTCAAGCCGTAGCGAGAATG	primer for amplification of 5' homology region of CH63R_14534
CK5272	CTGCATGGTCGTTTAAACATCGCTGGTGCGTGGTTC	primer for amplification of 5' homology region of CH63R_14534
CK5273	CACCAGCGATGTTTAAACGACCATGCAGACATACCTAGTGTTG	primer for amplification of the promotor region of CH63R_07477 with corresponding ends for chromosome 12 5' homology region
CK5277	GGGGACAACTTTGTATAATAAAGTTGTGCCAGGGCCGCTATACGAAGTG	primer for amplification of 3' homology region of CH63R_14406

CK5278	GGGGACAACTTTGTATAGAAAAGTTGTT CGTCCGCCGGAAGTGAGAAAT	primer for amplification of 3' homology region of CH63R_14406
CK5279	GGGGACAACTTTGTATAATAAAGTTGTAT GGGTGGCACCATCGACTGGG	primer for amplification of 3' homology region of CH63R_14534
CK5280	GGGGACAACTTTGTATAGAAAAGTTGTT GCTGATGGGGATCTGGAGAATGG	primer for amplification of 3' homology region of CH63R_14534
CK5295	CGATGGTCTTGTCTGGGTGAAC	primer for amplification of a fragment of CH63R_14381 on chromosome 11
CK5299	GTCGGAATCGAACCAACCGTC	primer for amplification of a fragment of CH63R_14406 on chromosome 11
CK5304	CCTCACGCTTCTCAACGC	primer for amplification of a fragment of CH63R_14635 on chromosome 12
CK5305	GTCAGCATCCGCCGTATAG	primer for amplification of a fragment of CH63R_14635 on chromosome 12
CK5327	CAGCGTCAGTCGTCCTCG	primer for verification of in locus integration of the 5' homology region into CH63R_14406
CK5328	GACCGTAACAGTAAGGAGTTGAC	primer for verification of in locus integration of the 3' homology region into CH63R_14406
CK5329	CAGTCCAGTCTGAGTCGGTG	primer for verification of in locus integration of the 5' homology region into CH63R_14534
CK5330	CAGCCTTGCGGTACATTCC	primer for verification of in locus integration of the 3' homology region into CH63R_14534
CK5393	GGGGACTGCTTTTTTGTACAAACTTGTCT GGCGTTGCCTGTTCTGTC	primer for amplification of a fragment of CH63R_14406 on chromosome 11
CK5449	ACATGGGACGTAACAGATAC	primer for the amplification of the JA-dependent PDF1.2a gene
CK5450	ATGGCTAAGTTTGCTTCCAT	primer for the amplification of the JA-

		dependent PDF1.2a gene
CK5451	CCTCACTTTGGCACATCCGA	primer for the amplification of the SA-dependent PR-1 gene
CK5452	AGGTGCTCTTGTTCTTCCCTC	primer for the amplification of the SA-dependent PR-1 gene
CK5453	CTGAACCTTCCTTGAGACGGA	primer for the amplification of the SA-dependent PR-2 gene
CK5454	CAAGGAGCTTAGCCTCACCAC	primer for the amplification of the SA-dependent PR-2 gene

Plasmids

Name	Synonym	Description	Marker (fungal)	Reference
pCK2275	pPK2	binary plasmid for generation of insertional mutants	hygR	(Covert et al., 2001)
pCK2650	pPN	binary plasmid; pPK2 derivative	natR	(Korn et al., 2015)
pCK3272	pOSCAR	binary vector for multisite gateway reaction		(Paz et al., 2011)
pCK3273	pA-Hyg-OSCAR	hygR donor vector for multisite gateway reaction	hygR	(Paz et al., 2011)
pCK3934		Bialaphos resistance (bar) donor vector for multisite gateway reaction	bar	JSchmidpeter, unpublished
pCK4122	pINLOCUS-mCherry	pINLOCUS-mCherry, pBluescript derivative		JSchmidpeter, unpublished

pCK5274	pINLOCUS-chr12-mCherry	pINLOCUS-mCherry with the 5' homology region of chromosome 12; pINLOCUS derivative		This study
pCK5293	pINLOCUS-chr11-mCherry	pINLOCUS-mCherry with the 5' homology region of chromosome 11; pINLOCUS derivative		This study
pCK5315	pchr11::mCherry	Binary plasmid for fluorescent tagging of chromosome 11; pOSCAR derivative	natR	This study
pCK5316	pchr11::mCherry	Binary plasmid for fluorescent tagging of chromosome 11; pOSCAR derivative	natR	This study
pCK5317	pPN-chr11::mCherry	binary plasmid for fluorescent tagging of chromosome 11; pPN derivative	bar/natR	This study
pCK5319	pPN-chr11::mCherry	binary plasmid for fluorescent tagging of chromosome 11; pPN derivative	bar/natR	This study
pCK5338	pchr12::mCherry	Binary plasmid for fluorescent tagging of chromosome 12; pOSCAR derivative	natR	This study
pCK5356	pPN-chr12::mCherry	binary plasmid for fluorescent tagging of chromosome 12; pPN derivative	bar/natR	This study

***C. higginsianum* strains**

Name	Genotype	transforming plasmid	parental strain	Reference
	C. higginsianum MAFF 305635			(O'Connell et al., 2004)

CY5976 *vir-49*	ATMT random insertional mutant	pPK2	MAFF 305635	(Korn et al., 2015)
CY6030 *vir-51*	ATMT random insertional mutant	pPK2	MAFF 305635	(Korn et al., 2015)
CY6153	*C. higginsianum* MAFF 305635 pma2Δ			(Korn et al., 2015)
CY7444 WT	Single colony of *C. higginsianum* MAFF 305635			this study
CY7466	*chr11::mcherry (natR)* #1	pCK5317	CY7444	this study
CY7467	*chr11::mcherry (natR)* #2	pCK5319	CY7444	this study
CY7527	*chr12::mcherry (natR)*	pCK5356	CY7444	this study
CY7548	*chr11*Δ single colony of CY7466 lacking chromosome 11 isolated by cell sorting		CY7466	this study
CY7550	*chr11*Δ single colony of CY7466 lacking chromosome 11 isolated by cell sorting		CY7466	this study
CY7551	*chr11*Δ single colony of CY7467 lacking chromosome 11 isolated by cell sorting		CY7467	this study
CY7554	*chr12*Δ single colony of CY7527 lacking chromosome 12 isolated by cell sorting		CY7527	this study
CY7581	*chr12*Δ chr11::mCherry	pCK5317	CY7554	this study
CY7589	*chr12*Δ*chr11*Δ single colony of CY7554 lacking chromosome 11 and 12 isolated by cell sorting		CY7581	this study

Plasmid constructions

Construction of plasmids for tagging chromosomes with an mCherry-fusion gene.

To genetically tag chromosome 11 with mCherry by homologous recombination, we constructed plasmid pCK5317 (see Figure below). This plasmid contains a bialaphos resistance gene (bar) in the region of homology for selection. To identify homologous recombinants after ATMT it further encodes a nourseothricin resistance gene (*nat*) as additional marker outside the region of homology. Homologous recombination of the construct disrupts the predicted endochitinase gene (CH63R_14406) located on the left arm of chromosome 11 by integration of the mCherry gene. We adopted the OSCAR strategy for generating the final plasmids (Paz et al., 2011). To get the 5' flank for homologous recombination in *C.higginsianum*, the NEBuilder ® HiFi DNA Assembly kit (New England Biolabs, #E2621) was used to assemble an 0.75 kb fragment of the endochitinase gene (amplified with CK5267 and CK5268), a 0.8 kb region containing the *Ch* TEF1 promoter (CH63R_07477, amplified with primers CK5269 and CK5270), and the vector pCK4122 (linearized with MssI). Plasmid pCK4122 contains the open reading frame of mCherry followed by a 200 bp polyA-site from the pyruvate kinase gene CH63R_14178 flanked by attachment sites attB2r (upstream of mCherry) and attB1r (downstream of the polyA site). The unique restriction site MssI is located at the ATG start codon of mCherry. The 5' flanking region, the TEF1 promotor fragment and the MssI digested mCherry donor vector (pCK4122) were assembled by NEBuilder ® HiFi DNA Assembly according to manufacturer instructions. The resulting plasmid (pCK5293) was digested with KpnI and BamHI to excise the 5' homology region fused to the mCherry expression construct. This fragment was used for one step gateway cloning together with the

corresponding 3' homology region (primers CK5277/CK5278, terminated by attB3 and attB4), pA-BAR-OSCAR and T-DNA vector pOSCAR (Paz et al., 2011) to produce a homology cassette with a bialaphos resistance gene. The resulting binary vector (pCK5315, pCK5316) was cleaved with Acc65I/NotI to excise the whole cassette. 5' overhangs were filled in with Klenow Fragment (Thermo Fischer, #EP0051). The resulting fragment was cloned into vector pPN (pCK2650, digested with MssI) to obtain binary vector pCK5317 (and pCK5319) with two resistance genes. One resistance gene (bar) located between 5' and 3' homology flank and the other (nat) outside the homology region. To tag chromosome 12 with mCherry, we constructed plasmid PCK5356 applying the same steps using the respective primers shown in the figure below.

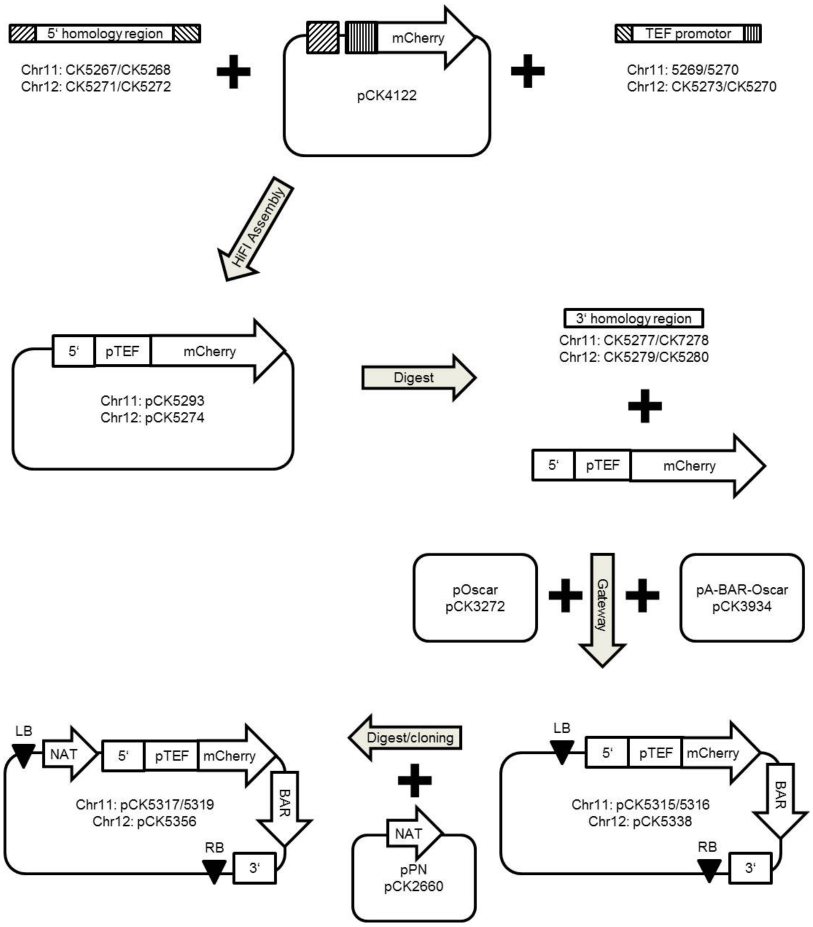

Figure: Construction of plasmids pCK5317 and pCK5356 for tagging chromosome 11 and chromosome 12 with mCherry fusions

References

Covert, S.F., Kapoor, P., Lee, M.H., Briley, A., and Nairn, C.J. (2001). Agrobacterium tumefaciens-mediated transformation of Fusarium circinatum. *Mycological Research* 105**,** 259-264. doi: Doi 10.1017/S0953756201003872.

Korn, M., Schmidpeter, J., Dahl, M., Muller, S., Voll, L.M., and Koch, C. (2015). A Genetic Screen for Pathogenicity Genes in the Hemibiotrophic Fungus Colletotrichum higginsianum Identifies the Plasma Membrane Proton Pump Pma2 Required for Host Penetration. *PLoS One* 10(5)**,** e0125960. doi: 10.1371/journal.pone.0125960.

O'Connell, R., Herbert, C., Sreenivasaprasad, S., Khatib, M., Esquerré-Tugayé, M.-T., and Dumas, B. (2004). A novel Arabidopsis-Colletotrichum pathosystem for the molecular dissection of plant-fungal interactions. *Mol Plant Microbe Interact* 17(3)**,** 272-282. doi: 10.1094/MPMI.2004.17.3.272.

Paz, Z., Garcia-Pedrajas, M.D., Andrews, D.L., Klosterman, S.J., Baeza-Montanez, L., and Gold, S.E. (2011). One step construction of Agrobacterium-Recombination-ready-plasmids (OSCAR), an efficient and robust tool for ATMT based gene deletion construction in fungi. *Fungal Genet Biol* 48(7)**,** 677-684. doi: 10.1016/j.fgb.2011.02.003.
