# Supplementary material for: A Dispensable Chromosome Is Required for Virulence in the Hemibiotrophic Plant Pathogen Colletotrichum higginsianum
Source: Front Microbiol. 2018 May 18;9:1005. doi: 10.3389/fmicb.2018.01005 (PMC5968395; doi:10.3389/fmicb.2018.01005)
Supplement: Supplementary file 3 [file Data_Sheet_2.PDF]

Supplementary data 2

DNA sequencing of *C. higginsianum* strain MAFF 305635 and *vir* mutants

In order to identify alterations in *vir-49* and *vir-51*, genomic DNA of the mutants and the WT strain MAFF 305635 were sequenced and compared to the reference genome assembly of IMI 349063 (Dallery et al., 2017) to detect mutations and to also determine polymorphisms between coding regions of the reference genome and the MAFF 305635 strain used for this study.

The raw sequencing files (see Materials and Methods) were deposited into the Sequence Read Archive (SRA) at NCBI (accession number of bioproject: PRJNA427316) (.fastq accessions: SAMN08226879, SAMN08226880, SAMN08226881). Sequencing reads mapped to the reference genome assembly of strain IMI 349063 (accession: GCA_001672515.1) were compressed into BAM files and deposited into the SRA.

***Vir-49* and *vir-51* lack chromosome 11**

The number of sequencing reads mapping to each chromosome and gene was calculated for each mutant and the MAFF 305635 WT strain using BEDTools' (Quinlan and Hall, 2010) coverage subcommand. The number of reads for each chromosome and each gene is given in Table S1 and Table S2, respectively.

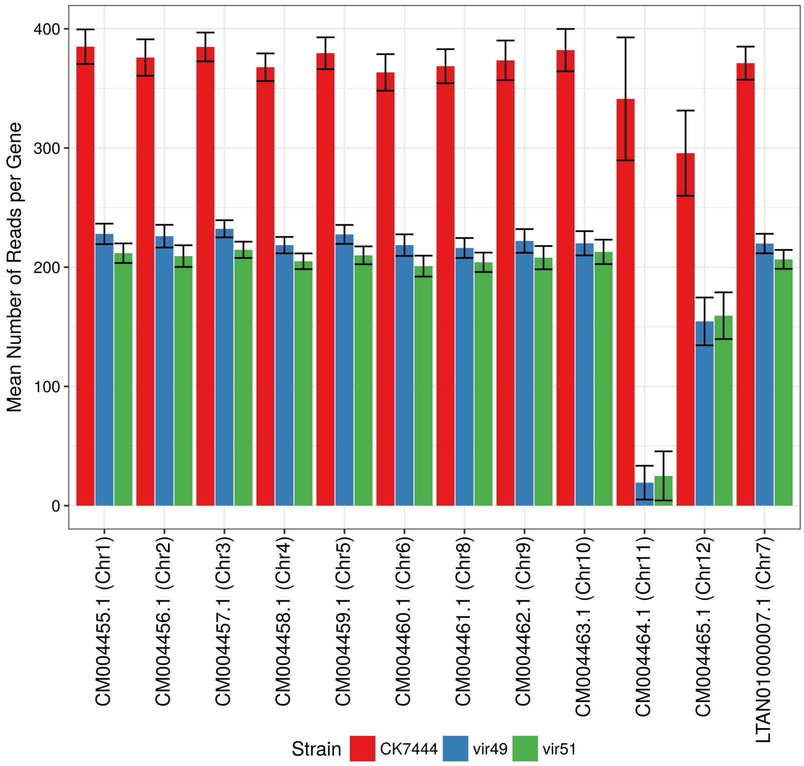

Fig. Mean number of reads for WT (CK7444) and vir mutant DNAs mapping to individual chromosomes.

The median of reads per gene was 318 for the WT DNA; 189 for the vir-49 DNA and 174 for the vir-51 DNA (see also Table S2). Genes with less than 10 mapped reads are shown in Table S3. In the WT 95 genes were supported by less than 10 reads and may potentially be absent in strain MAFF 305635. For mutants *vir-49* and *vir-51* the picture was the same except for chromosome 11 (Table S3).

For MAFF 305635 WT, 1.3% of all mapped reads were mapped to genes on chromosome 11. In contrast, for vir-49 and vir51 only 0.2 and 0.3% of all mapped reads, were mapped to genes on chromosome 11, indicating that the two mutants have lost the whole chromosome. This is also shown in the figure above that shows the average number of mapped reads per chromosome for each strain. The mapping

of vir-49 reads to chromosome 11 is shown in the figure below and indicates that most regions with high coverage (blue) largely coincide with transposons (purple).

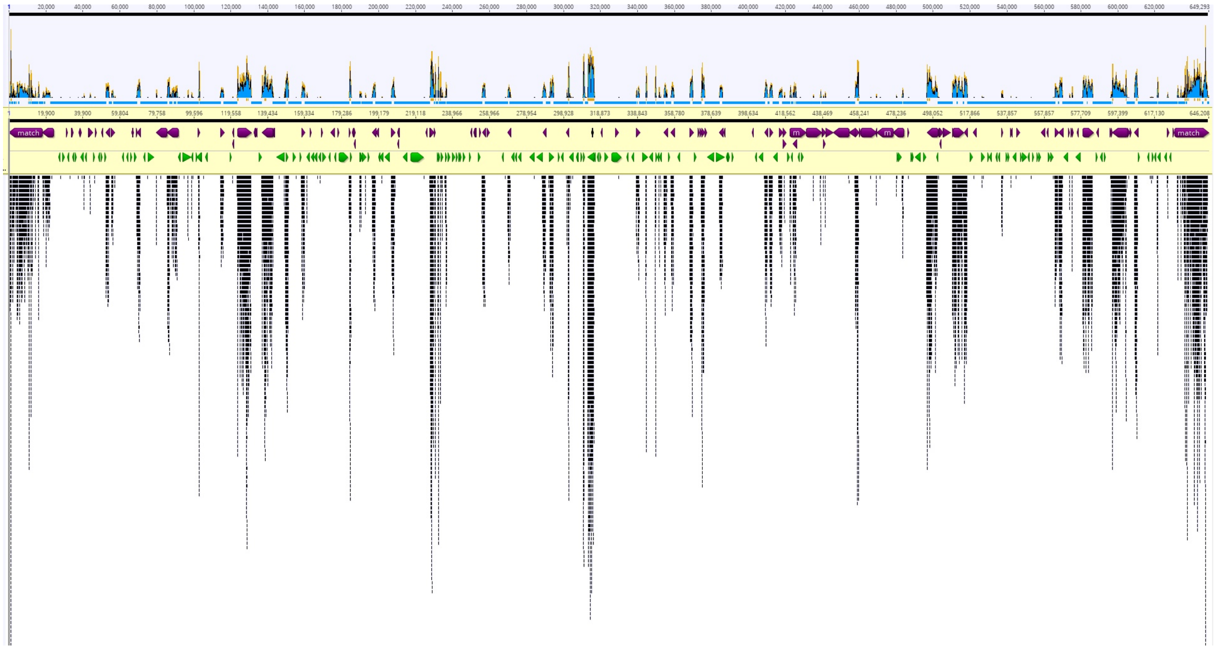

Fig. Reads from vir-49 DNA map mostly to transposable elements in chromosome 11.

Reads mapping to chromosome 11 in the mutants were in regions annotated as transposable elements except for twenty-three regions annotated as genes (CH63R_14399, CH63R_14400, CH63R_14402, CH63R_14408, CH63R_14411, CH63R_14419, CH63R_14420, CH63R_14421, CH63R_14429, CH63R_14430, CH63R_14431, CH63R_14432, CH63R_14447, CH63R_14450, CH63R_14454, CH63R_14455, CH63R_14456, CH63R_14463, CH63R_14465, CH63R_14467, CH63R_14472, CH63R_14489 and CH63R_14516). On closer inspection only six genes (CH63R_14408, CH63R_14419, CH63R_14430, CH63R_14454, CH63R_14455 and CH63R_14489) are covered with at least one read over the

whole coding region. For the remaining genes coverage ranged from 6% to 91% with a median of 44%. Most of the reads that were mapped to these twenty-three genes exhibit a relatively large number of differences compared to the reference sequence, suggesting they were either not correctly mapped, or quite different to the reference. For example CH63R_14455, a potential DNA repair helicase, had an identity of only 91%. In addition, most of these reads showed low mapping quality. For example, the average mapping qualities of the reads mapped to CH63R_14489 were 1.2 (*vir-49*) and 1.6 (*vir-51*). All of these reads almost certainly originate from regions of the genome different from chromosome 11 because according to the PFGE analysis chromosome 11 is absent in the two *vir* mutants and because most of the corresponding mapping of WT reads showed two different groups of reads for most of these gene IDs. One set of reads perfectly matched the reference gene, while the second set of reads matched the reads found in the *vir* mutants (see below). While it is clear that the additional reads in *vir-49* and *vir-51* are not derived from chromosome 11, the available sequencing data was not sufficient to identify from which of the other chromosomes in MAFF 305635 these ORFs originate from.

Genomic differences between MAFF 305635 and the reference genome assembly of IMI 349063

99% of the annotated reference genes were found in the MAFF 305635 sequencing reads. There are, however, notable differences between the two isolates. In the published reference genome of strain IMI 349063 chromosome 11 contains 646 mb and chromosome 12 has a size of 597 mb. The karyotype of MAFF 305635 showed

differences in the size of the two mini chromosomes. Chromosome 11 was lacking approximately 30 kb, while chromosome 12 was roughly 200 kb larger than the corresponding chromosome in the reference strain. The values from the PFGE must however be considered rough estimates.

Table: Size differences between IMI 349063 and MAFF 305635 dispensable chromosomes

	IMI 349063*	MAFF 305635**
chromosome 11	646 mb	620 mb
chromosome 12	597 mb	800 mb

*published sizes based on sequence assembly (Dallery et al., 2017) ** size estimated from pulsed field gel electrophoresis

The DNA sequencing supported the difference found for chromosome 11 by PFGE. There was a region of low sequencing coverage as shown below.

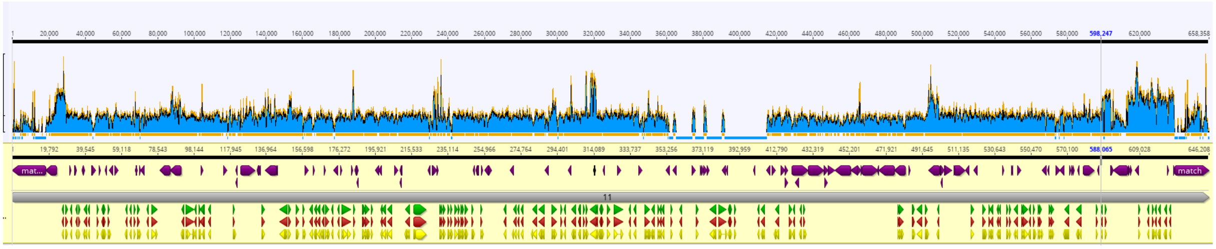

Fig.: Low coverage of MAFF 305635 reads in the 50 kb region of chromosome 11 between annotated genes CH63R_14468 and CH63R_14477.

The region encompasses nine potential genes (CH63R_14468 to CH63R_14477).

Some additional regions in which the two strains differ were identified by looking for segments of low coverage from the MAFF 305635 strain (Table S2).

These include small regions from the end of chromosome 2 and 3.50 kb from the left arm of chromosome 8, 50 kb from the left arm of chromosome 9 and a region of 40 kb on chromosome 4 (CH63R_05483 to CH63R_05497). Another region which is apparently absent from strain MAFF 305635 is a 74 kb segment in chromosome 6 that contains 20 genes in the reference assembly (CH63R_09609 to CH63R_09628).

Additional copies for some genes on chromosome 11

On chromosome 11 a second region of interest corresponds to some of the aforementioned genes (CH63R_14455, CH63R_14408, CH63R_14454, CH63R_14447, CH63R_14419, and CH63R_14516). For these we noticed that two different kinds of reads from the WT mapped there. For ID CH63R_14455 part of the DNA sequencing alignment is shown as an example in the figure below.

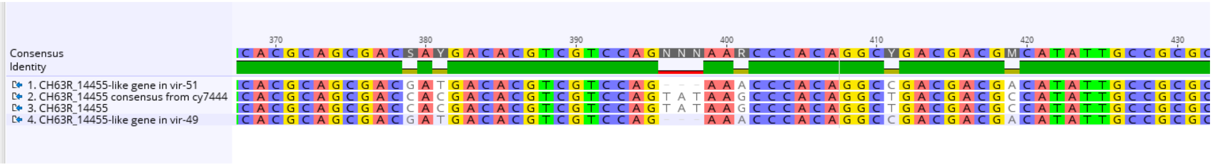

Fig.: A additional CH63R_14455 –like gene may be present in MAFF 305635 and the *vir* mutants *vir-49* and *vir-51*. Software Geneious 10.2 (https://www.geneious.com) was used to align and display the DNA alignment.

By PCR analysis we confirmed for CH63R_14455 that both variants are present in MAFF 305635 DNA, while only the CH63R_14455 – like DNA is found in *vir-49* DNA. Furthermore, the CH63R_14455 – like gene appears to be specific for MAFF 305635

as the respective PCR-fragment was not amplified with DNA from the reference genome IMI 349063. Interestingly, the CH63R_14455-like gene potentially encodes a protein with 85 % identity to CH63R_14455, lacking introns and predicted to encode a protein annotated as a 1081 amino acid protein with similarity to DNA repair helicases while protein blastp and tblastn showed that significant hits (e< 5e-8) were only found in *Colletotrichum* species.

Interestingly, another of the genes with an extra copy from other regions of the genome corresponds to CH63R_14516. It encodes EC12a which is a potential effector protein with two copies on chromosome 11 of the reference encoded by CH63R_14516 and CH63R_14389. This may indicate that a third copy of this gene is present at a different location in MAFF 305635.

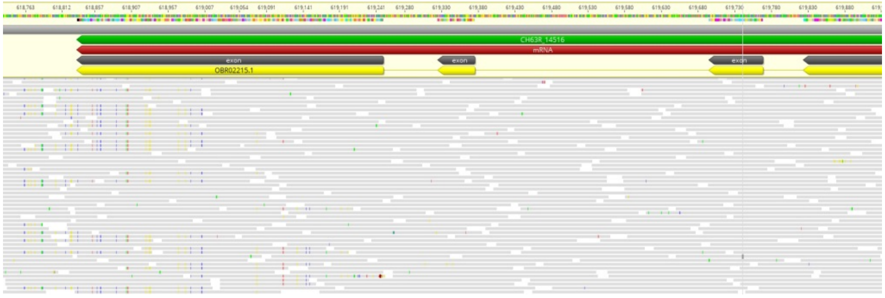

Fig: Reads specific to MAFF 305635 with similarity to CH63R_14516, encoding the potential effector EC12a.

Variant calling, filtering and annotation between IMI 349063 and MAFF 305635

Duplicate reads were marked using Picard's MarkDuplicates tool (http://broadinstitute.github.io/picard, v2.15.0) with the following command line parameters: VALIDATION_STRINGENCY=LENIENT

REMOVE_DUPLICATES=false, and removed with samtools (-F 1024). Duplicates are usually artifacts due to the PCR cycles during library preparation. Keeping duplicates for downstream analysis might lead to propagation of sequencing errors and false positive variants. In addition, only reads with a mapping quality higher than 20 (-q 20) were retained for further analysis. Indels were realigned with GATK's (v3.4-46) IndelRealigner (McKenna et al., 2010). Indel realignment is a step in which reads present in the region of an indel are realigned to minimize the number of variants explaining the observed reads. Variants were called using SAMtools's (v1.6,) (Li et al., 2009) subcommand "mpileup" with default parameters and VarScan's (v2.3.9) (Koboldt et al., 2009) subcommand "mpileup2cns" with parameter "--variants". Variants were called with VarScan (v2.3.9) using the subcommand "mpileup2cns" with parameter "--variants". "Pileup2cns --variants" reports all positions at which a SNP or an indel was called. We kept the default values for all remaining parameters, implying that at least eight reads are required at a position to make a call, at least two supporting reads are required to support any variant call, and variants are called with a P-value threshold of 0.01.

Variants were annotated using snpEff (v4.3t) (Cingolani et al., 2012) with parameters "-formatEff", "-stat", and "-csvStats". Genome annotation for *Colletotrichum higginsianum* IMI 349063 was retrieved from NCBI in GFF3 format. VCF output files were used to classify variants on the basis of their occurrence in the intergenic, intronic, and exonic regions. snpEff annotates variants with terms in the sequence ontology database (http://www.sequenceontology.org, (Eilbeck et al., 2005). Variants annotated with the term "coding_sequence_variant" (SO:0001580) were considered protein-coding variants and evaluated as classified synonymous or non-synonymous variants according to their effects (Table S4). On average the ratio of synonymous to missense variants was 1,3. Missense variants are defined as sequence variant, that

changes one or more bases, resulting in a different amino acid sequence but where the length is preserved. Note that not all variants annotated as “coding_sequence_variant” can be classified as synonymous or missense (e.g., “start_lost”, “stop_gained” and “splice_acceptor_variant” are types of “coding_sequence_variant” but neither “synonymous_variant” nor “missense_variant”).Protein-coding variants were next filtered to a variant frequency of 70%. Data for the variant calling are shown in Table 1 and Table S4.

References

Cingolani, P., Platts, A., Wang le, L., Coon, M., Nguyen, T., Wang, L., et al. (2012). A program for annotating and predicting the effects of single nucleotide polymorphisms, SnpEff: SNPs in the genome of Drosophila melanogaster strain w1118; iso-2; iso-3. *Fly (Austin)* 6(2)**,** 80-92. doi: 10.4161/fly.19695.

Dallery, J.F., Lapalu, N., Zampounis, A., Pigne, S., Luyten, I., Amselem, J., et al. (2017). Gapless genome assembly of Colletotrichum higginsianum reveals chromosome structure and association of transposable elements with secondary metabolite gene clusters. *BMC Genomics* 18(1)**,** 667. doi: 10.1186/s12864-017-4083-x.

Eilbeck, K., Lewis, S.E., Mungall, C.J., Yandell, M., Stein, L., Durbin, R., et al. (2005). The Sequence Ontology: a tool for the unification of genome annotations. *Genome Biol* 6(5)**,** R44. doi: 10.1186/gb-2005-6-5-r44.

Koboldt, D.C., Chen, K., Wylie, T., Larson, D.E., McLellan, M.D., Mardis, E.R., et al. (2009). VarScan: variant detection in massively parallel sequencing of individual and pooled samples. *Bioinformatics* 25(17)**,** 2283-2285. doi: 10.1093/bioinformatics/btp373.

Li, H., Handsaker, B., Wysoker, A., Fennell, T., Ruan, J., Homer, N., et al. (2009). The Sequence Alignment/Map format and SAMtools. *Bioinformatics* 25(16)**,** 2078-2079. doi: 10.1093/bioinformatics/btp352.

McKenna, A., Hanna, M., Banks, E., Sivachenko, A., Cibulskis, K., Kernytsky, A., et al. (2010). The Genome Analysis Toolkit: a MapReduce framework for analyzing next-generation DNA sequencing data. *Genome Res* 20(9), 1297-1303. doi: 10.1101/gr.107524.110.

Quinlan, A.R., and Hall, I.M. (2010). BEDTools: a flexible suite of utilities for comparing genomic features. *Bioinformatics* 26(6), 841-842. doi: 10.1093/bioinformatics/btq033.
