# Supplementary figures and images for: A Dispensable Chromosome Is Required for Virulence in the Hemibiotrophic Plant Pathogen Colletotrichum higginsianum
Source: Front Microbiol. 2018 May 18;9:1005. doi: 10.3389/fmicb.2018.01005 (PMC5968395; doi:10.3389/fmicb.2018.01005)

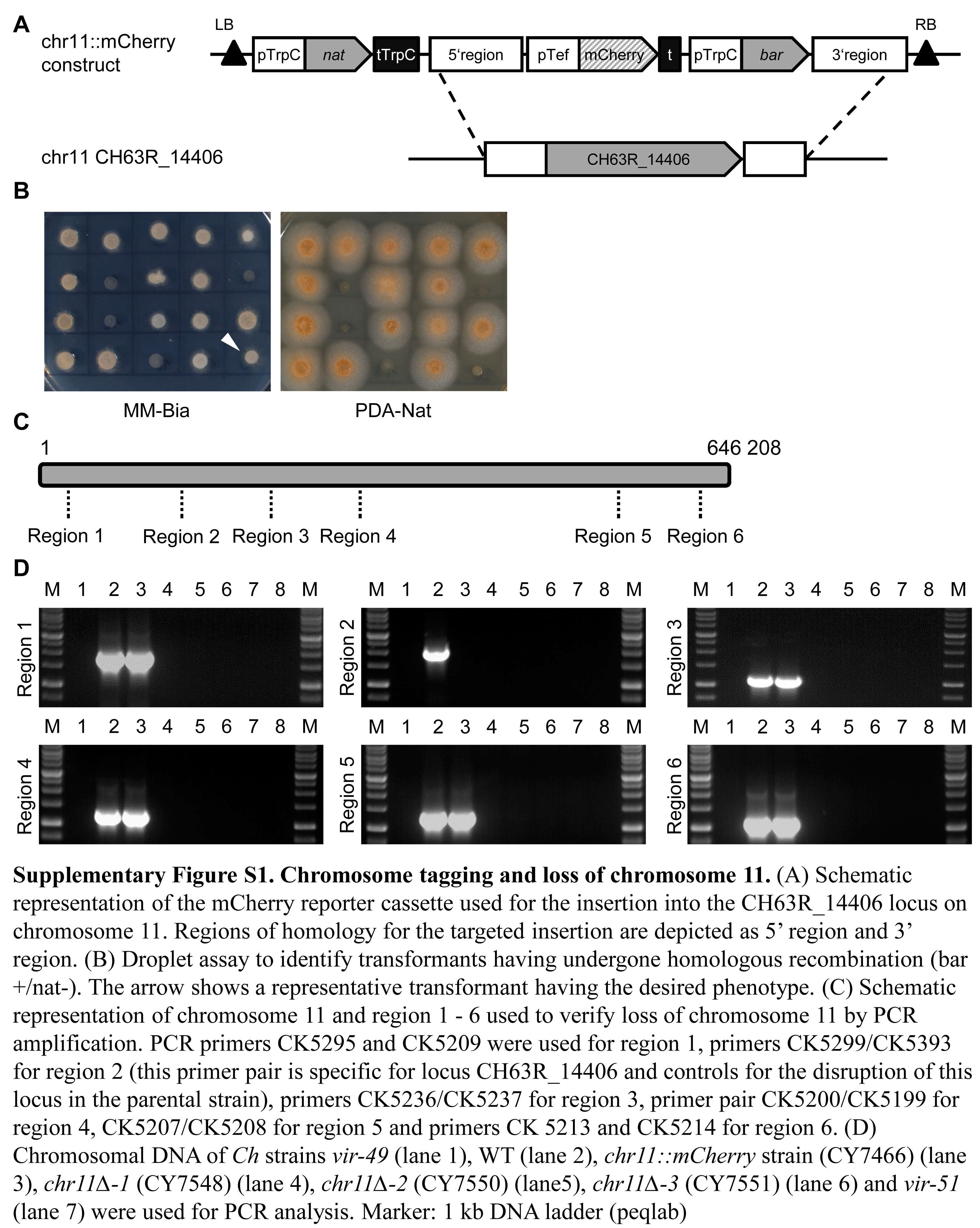

Supplement: Supplementary file 4 [file Image_1.TIF]

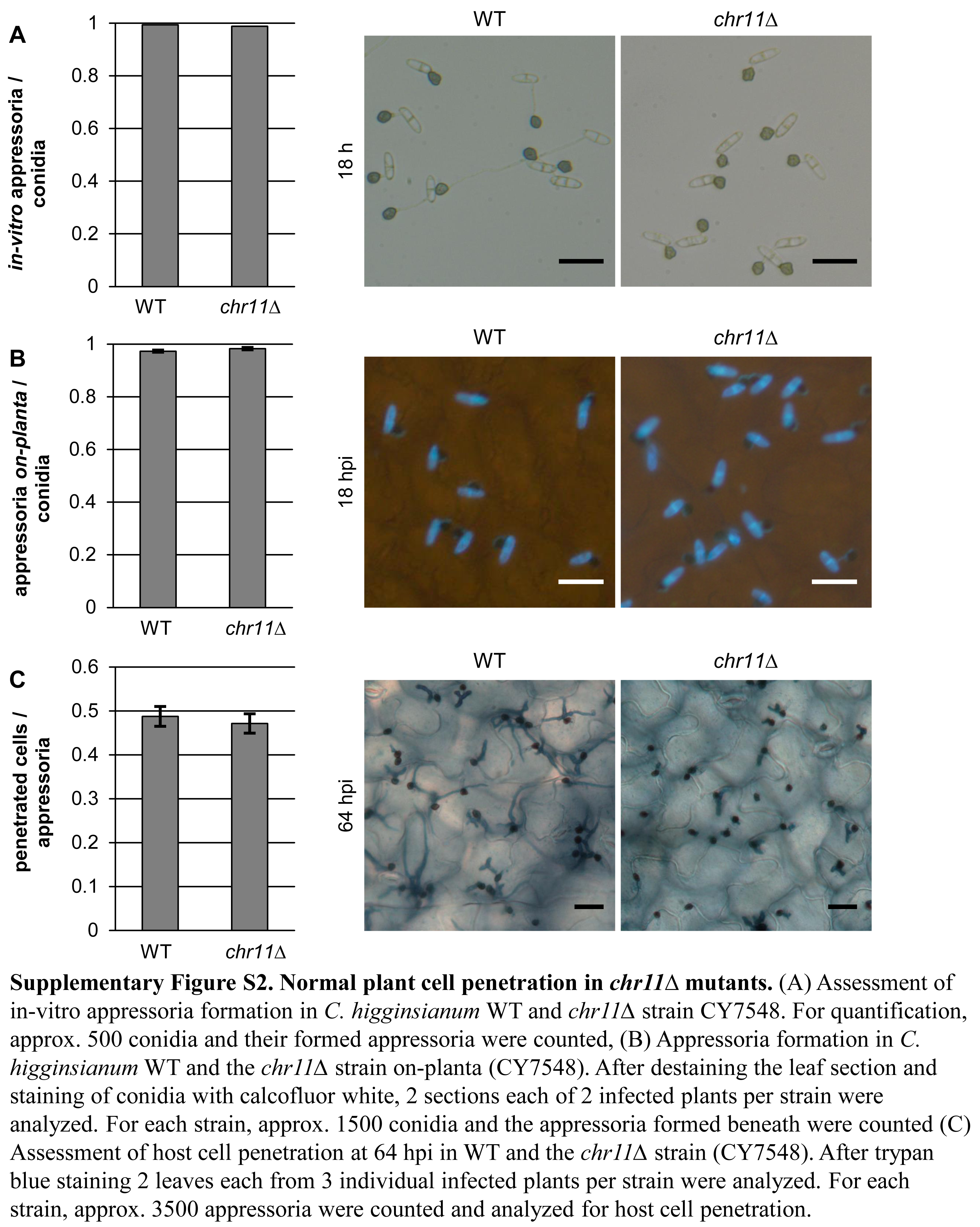

Supplement: Supplementary file 5 [file Image_2.TIF]

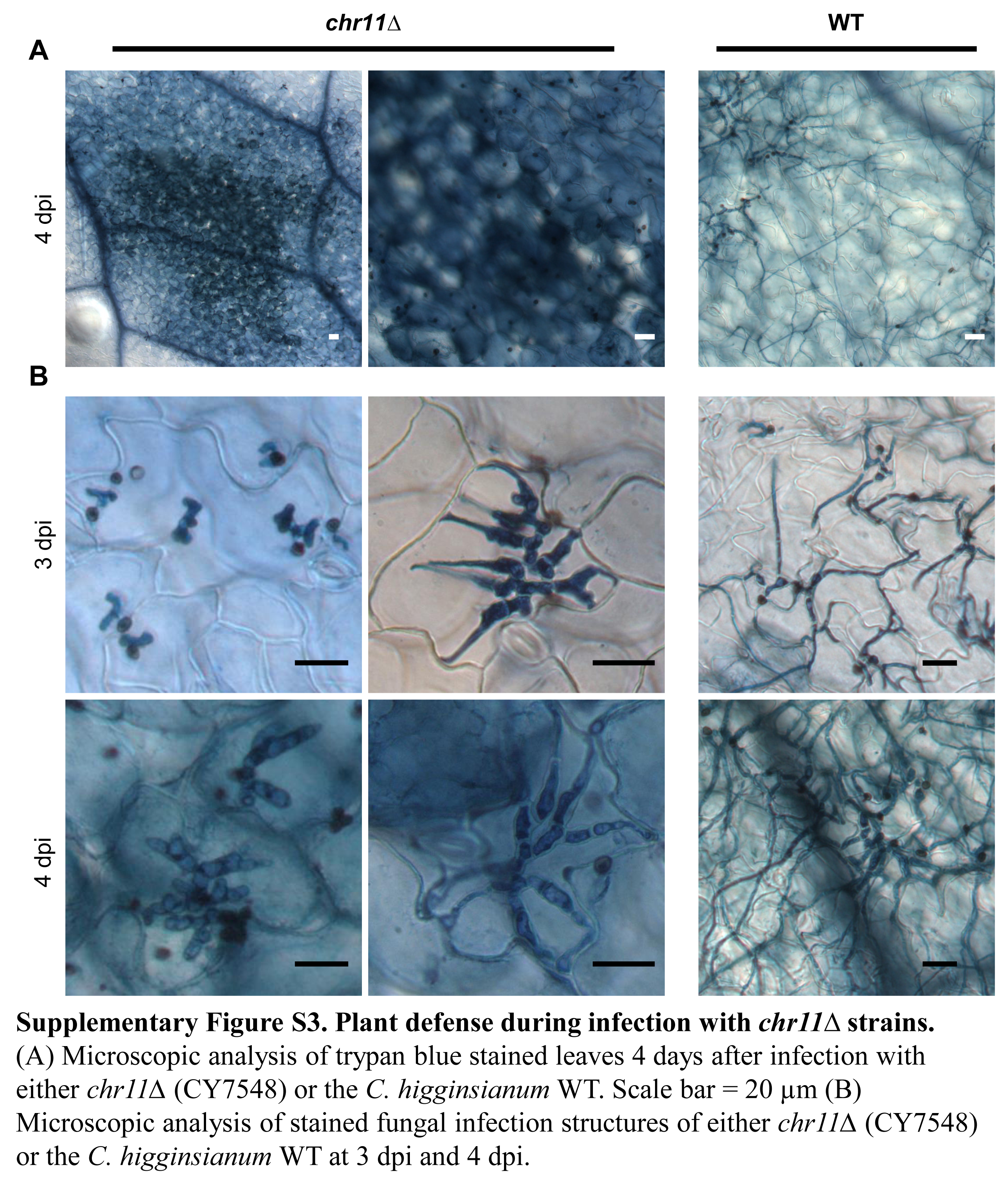

Supplement: Supplementary file 6 [file Image_3.TIF]

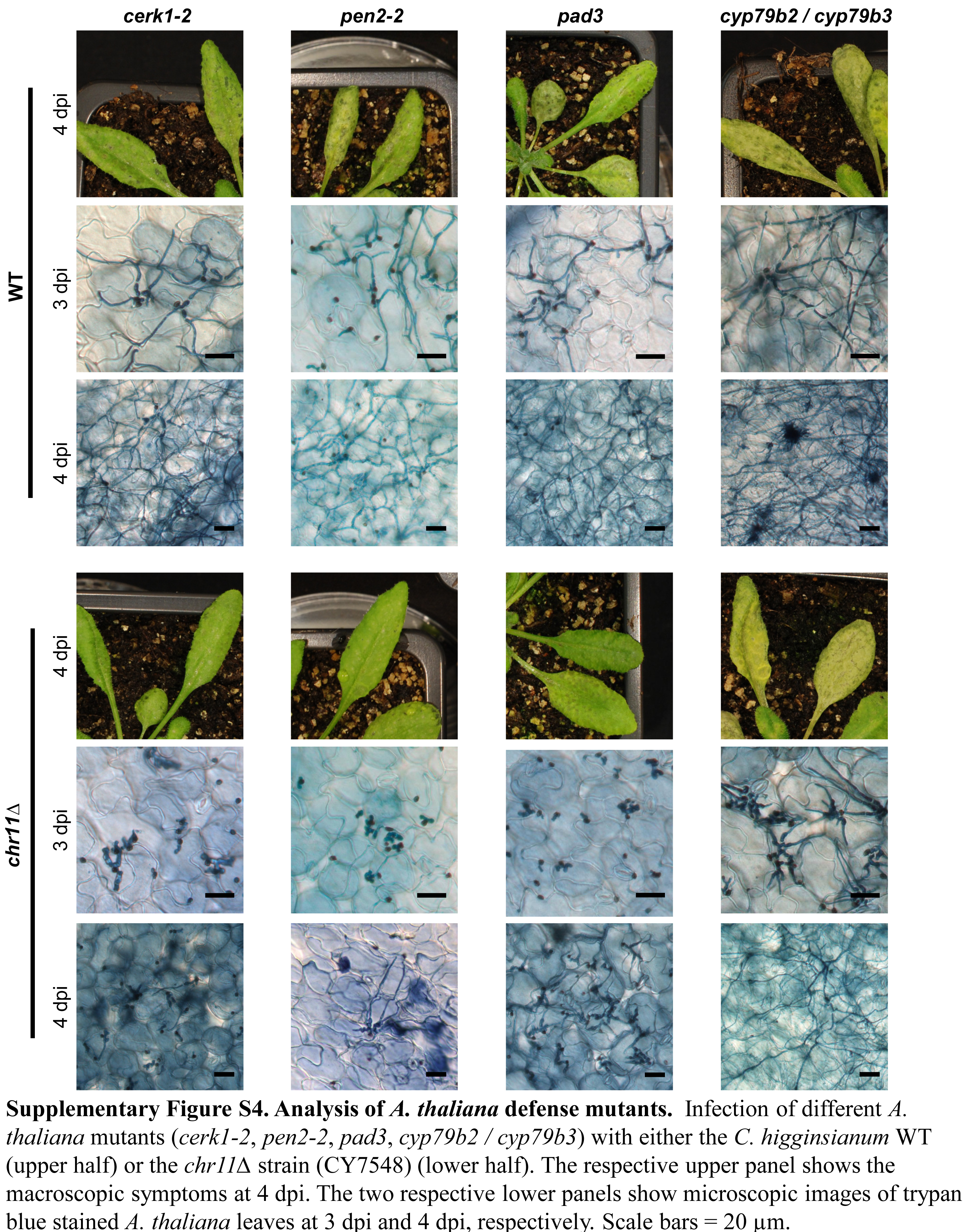

Supplement: Supplementary file 7 [file Image_4.TIF]

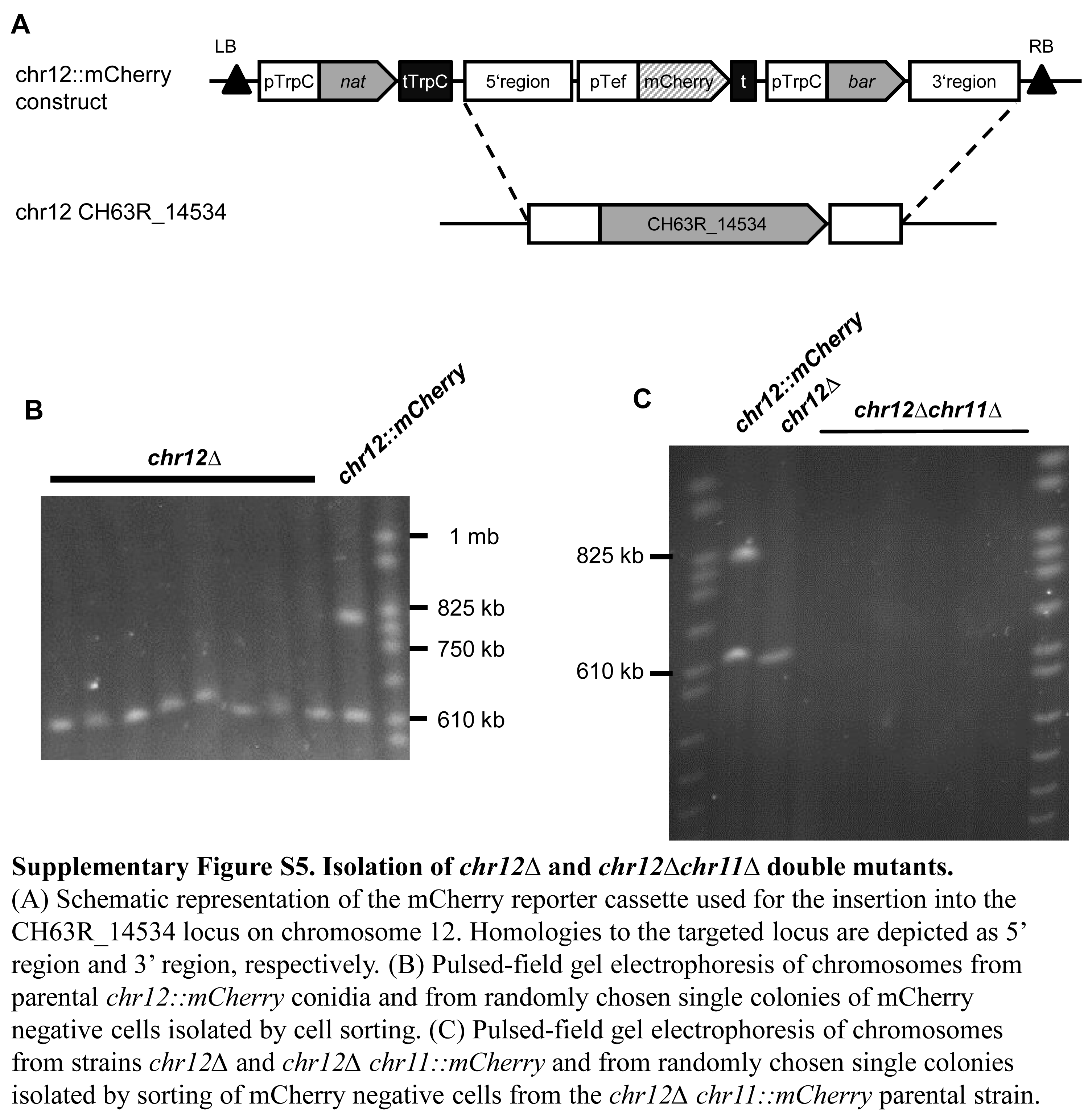

Supplement: Supplementary file 8 [file Image_5.TIF]
